# Weak CO binding sites induced by Cu–Ag interfaces promote CO electroreduction to multi-carbon liquid products

**DOI:** 10.1038/s41467-023-36411-5

**Published:** 2023-02-08

**Authors:** Jing Li, Haocheng Xiong, Xiaozhi Liu, Donghuan Wu, Dong Su, Bingjun Xu, Qi Lu

**Affiliations:** 1 State Key Laboratory of Chemical Engineering, Department of Chemical Engineering, Tsinghua University, 100084 Beijing, China; 2 College of Chemistry and Molecular Engineering, Peking University, 100871 Beijing, China; 3 Beijing National Laboratory for Condensed Matter Physics, Institute of Physics, Chinese Academy of Sciences, 100190 Beijing, China

**Keywords:** Electrocatalysis, Electrocatalysis, Electrocatalysis, Surface spectroscopy, Electrocatalysis

## Abstract

Electrochemical reduction of carbon monoxide to high-value multi-carbon (C_2+_) products offers an appealing route to store sustainable energy and make use of the chief greenhouse gas leading to climate change, i.e., CO_2_. Among potential products, C_2+_ liquid products such as ethanol are of particular interest owing to their high energy density and industrial relevance. In this work, we demonstrate that Ag-modified oxide-derive Cu catalysts prepared via high-energy ball milling exhibit near 80% Faradaic efficiencies for C_2+_ liquid products at commercially relevant current densities (>100 mA cm^−2^) in the CO electroreduction in a microfluidic flow cell. Such performance is retained in an over 100-hour electrolysis in a 100 cm^2^ membrane electrode assembly (MEA) electrolyzer. A method based on surface-enhanced infrared absorption spectroscopy is developed to characterize the CO binding strength on the catalyst surface. The lower C and O affinities of the Cu–Ag interfacial sites in the prepared catalysts are proposed to be responsible for the enhanced selectivity for C_2+_ oxygenates, which is the experimental verification of recent computational predictions.

## Abstract (web summary)

Here, the authors demonstrate a Cu-based catalyst with Cu–Ag interfacial sites, which favor oxygenate over alcohol production in CO_2_ electroreduction. Near 80% selectivity for multi-carbon liquid products in a 100 cm^2^ membrane electrode assembly electrolyzer is exhibited over 100 h.

## Introduction

The electrochemical reduction of carbon dioxide to commodity chemicals and fuels has become a promising approach to mitigate CO_2_ emissions and store intermittent renewable energy^1,2^. To improve the efficiency of CO_2_ reduction reaction (CO_2_RR) to highly valuable multi-carbon products, substantial progress has been made in the development of gas-diffusion type flow cells (e.g., microfluidic reactors and membrane electrode assembly electrolyzers) to improve the mass transport of CO_2_^3–8^. A reservoir of flowing highly alkaline electrolytes is typically used in these electrolyzers, with the purpose of increasing the full-cell energy efficiency by reducing the voltage required to drive the coupled anodic oxygen evolution reaction^9^. However, the use of highly alkaline electrolytes inevitably leads to electrolyte degradation and coking of the electrode due to the carbonate formation caused by the chemical reaction of CO_2_ and OH^−^, which poses great challenges to the improvement of carbon efficiency to support commercial applications^10^. In a few recent studies, the carbonate formation problem has been effectively reduced by conducting CO_2_ electrolysis in strong acid media or directly implementing carbonate to CO_2_RR products^11–13^. Despite the progress, the energy efficiency for the multi-carbon (C_2+_) products is below expectation due to their low total Faradaic efficiency (less than 50%) achieved at those conditions^12,13^. The use of CO instead of CO_2_ as the feedstock offers a means to address these challenges resulting from the undesired side reaction or low selectivity. In light of the comparatively mature electrochemical conversion of CO_2_ to CO in non-alkaline conditions (e.g., in strong acid media or using solid oxide electrochemical cell) with high efficiency^11,14^, the tandem strategy in which CO_2_ is first reduced to CO followed by further reducing CO to multi-carbon products shows significant promise^15,16^.

In previous studies of CO reduction reaction (CORR), it has been demonstrated that Cu-based catalysts are capable of converting CO to high-value multi-carbon products which are composed of a mixture of gaseous ethylene and liquid C_2+_ products^17–24^. The latter typically including ethanol, acetate, and n-propanol is of particular interest because these liquid products are in high energy densities by volume and are more convenient for storage and transport than gaseous products^25^. Many efforts have been dedicated to the improvement of C_2+_ product formations, including engineering Cu facet^20,23^, structuring grain boundaries^17^, and alloying^22^, etc. However, to date, most of the state-of-the-art catalysts favor ethylene production over liquid C_2+_ products^8,18,20,21,26,27^. In spite of the improved Faradaic efficiency up to 70% towards C_2+_ liquid products of oxide-derived Cu at very low overpotentials (>−0.3 V_RHE_)^17,28^, the corresponding Faradaic efficiency reduces to below 40% when operating at more negative potentials with commercial viable current densities (>100 mA cm^−2^)^18^. A recent study shows that high roughness factor Cu electrodes are capable of achieving almost full selectivity towards C_2+_ liquid products at low overpotentials^19,29^. However, the corresponding current densities of these electrodes are too low (<1 mA cm^−2^) to fulfill the requirements of commercial applications. Therefore, it remains a challenge to develop electrocatalysts that can selectively produce C_2+_ liquid products with commercially relevant current densities.

In this work, we report a high-energy ball milling process for the preparation of an Ag-modified oxide-derived Cu catalyst, which exhibits a total selectivity of nearly 80% for C_2+_ liquid products at commercially viable current densities in the CORR. This result represents a significant improvement in performance over the state-of-the-art catalysts for the production of C_2+_ liquid products. By employing a custom-designed two-compartment electrochemical spectroscopic cell, we estimate the adsorbed CO (CO_ad_) desorption rate constant on various catalyst surfaces and find the Ag-modified oxide-derived Cu catalyst surface gives an increased number of weakly bound CO_ad_. Combining the structural characterization and the CO/Ar switching experiments, we show that the weakly bound CO_ad_ is correlated with the Cu–Ag interfacial sites and is responsible for the enhanced C_2+_ liquid product formation. The improved performance could be due to the lower C and OH affinities of the Cu–Ag interfacial, which have been predicted to favor oxygenates over ethylene^30,31^. The ball milling time-dependent study highlights the drastic impact of the structural properties of Ag-modified oxide-derived Cu catalysts on C_2+_ liquid product selectivity. The long-time-milled catalysts present more abundant and stable Cu–Ag phase boundaries, which may be a potential cause leading to higher activity and selectivity towards C_2+_ liquid products compared with those reported CuAg bimetallic catalysts prepared via other methods, such as galvanic replacement^29,32,33^, co-electrodeposition^34^, and high-temperature shock^22^.

## Results

### CORR performance of the Ag-modified Cu catalysts

The Ag-modified Cu catalysts were prepared by high-energy ball milling CuO particles with Ag powders with different atomic ratios of Cu:Ag (0.9:0.1, 0.8:0.2, 0.7:0.3, 0.5:0.5) (see “Methods” for details). CuO, instead of metallic Cu, is chosen as the precursor for ball milling to avoid cold welding that can severely increase the size and reduce the homogeneity of the synthesized materials^35,36^. The as-prepared catalysts were supported onto the microporous layer of carbon fiber paper as gas-diffusion type electrodes for reactivity evaluations in 1 M KOH in a three-compartment microfluidic cell. Prior to reactivity measurements at different potentials, electrodes were pretreated with an in situ electroreduction at a constant current density of −5 mA cm^−2^ for 5 mins to convert CuO into metallic Cu (i.e., oxide-derived Cu). The resulting catalysts are denoted as Cu(OD)_1__−__*x*_Ag_*x*_ (*x* = 0, 0.1, 0.2, 0.3, 0.5) in which Cu(OD) stands for oxide-derived Cu.

**Fig. 1:**
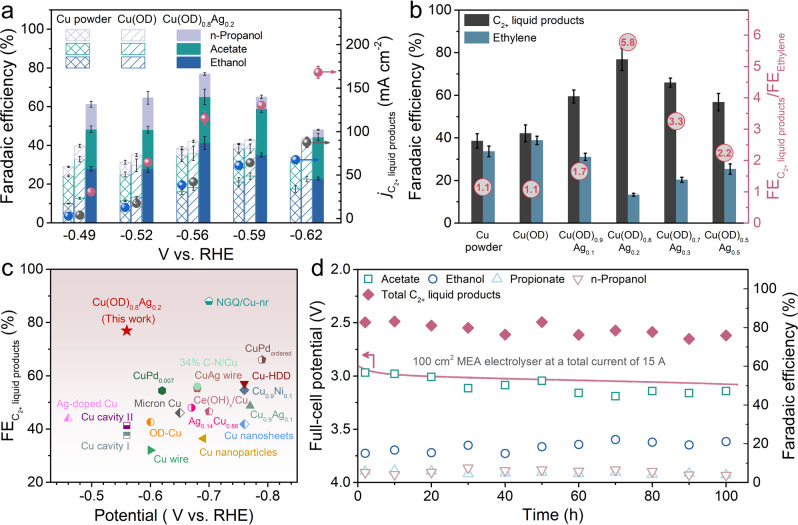
Carbon monoxide electroreduction performance. **a** Faradaic efficiency and total current densities of C_2+_ liquid products (acetate, ethanol, and n-propanol) on polycrystalline Cu powder, Cu(OD), and Cu(OD)_0.8_Ag_0.2_ catalysts at various applied potentials in 1 M KOH. **b** Faradaic efficiency of C_2+_ liquid products and ethylene on all different catalysts at –0.56 ± 0.01 V_RHE_. Numbers in the red circles show the corresponding Faradaic efficiency ratio of C_2+_ liquid products to ethylene. **c** Comparison of the maximum C_2+_ liquid products FE versus applied potential between Cu(OD)_0.8_Ag_0.2_ in this work and state-of-the-art catalysts in literature at a commercially viable current density (>100 mA cm^−2^) in CORR and CO_2_RR (see Supplementary Table 1 for more details). **d** Full-cell voltage and the Faradaic efficiencies for C_2+_ liquid products in 103-h electrolysis at a constant current of 15 A in a 100 cm^2^ custom-designed MEA electrolyzer. The error bars represent standard deviations from at least three independent measurements.

The partial current densities and Faradaic efficiencies (denoted as FE) for each catalyst are provided in Supplementary Fig. 1. The Cu(OD) catalyst and polycrystalline Cu power catalyst exhibited near identical product distributions with gaseous ethylene as the major product in the potential window investigated, which is consistent with previous literatures^8,18,27^. Cu(OD) was reported to be more selective to C_2+_ product formations at smaller overpotentials^17,18^. The FE towards CORR products for all catalysts increased with the negatively biased potential with a maximum FE achieved at approximately −0.55 to −0.6 V_RHE_, while a further decrease of electrode potential reduces the CORR FE due to the enhanced HER. Methane is a minor product in the potential range of this study (FE < 3%) and thus is not discussed further. Remarkably, the FE of C_2+_ liquid products, i.e., ethanol, acetate, and n-propanol, substantially increased in all Cu(OD)_1__−__*x*_Ag_*x*_ catalysts, accompanied by the concomitantly decreased FE of ethylene (Fig. 1a, b and Supplementary Fig. 2). The optimal selectivity for C_2+_ liquid products was achieved in Cu(OD)_0.8_Ag_0.2_, exhibiting an FE close to 80% at −0.56 V_RHE_, which was a near twofold improvement compared to that in Cu(OD) and polycrystalline Cu powder catalysts (Fig. 1a). The FEs of C_2+_ liquid products and ethylene, as well as their ratios at −0.56 ± 0.01 V_RHE_ were plotted for all catalysts (Fig. 1b). The FE ratio of C_2+_ liquid products/ethylene increased as the increase of Ag content with an optimal value of 5.8 achieved at Cu(OD)_0.8_Ag_0.2_, while slightly decreased with further increases of Ag. The formation rate of C_2+_ liquid products was also enhanced with the addition of Ag (Fig. 1a). To the best of our knowledge, the selectivity of C_2+_ liquid products achieved on the Cu(OD)_0.8_Ag_0.2_ is among the highest values reported to date for both CORR and CO_2_RR conducted at a commercially viable rate (>100 mA cm^−2^) (Fig. 1c and Supplementary Table 1). The Cu(OD)_0.8_Ag_0.2_ catalyst was evaluated in a 100 cm^2^ custom-designed membrane electrode assembly electrolyzer (see “Methods” and Supplementary Fig. 3 for details) to demonstrate it is able to retain the high performance as the electrode area increases. As shown in Fig. 1d, an FE of up to 83% for C_2+_ liquid products including acetate, ethanol, propionate, and n-propanol at a constant current of 15 A was achieved. The MEA system maintained a high C_2+_ liquid products FE of ~80% and a stable full-cell voltage of −2.98 ± 0.09 V during a 103-h CO electrolysis. In contrast, Cu(OD) exhibited significantly less C_2+_ liquid products FE (Supplementary Fig. 4). Moreover, a high single-pass CO conversion rate of ~76% was also achieved. These results clearly demonstrate that the Cu(OD)_0.8_Ag_0.2_ catalyst is able to perform at the same high level when the electrode area increases to 100 cm^2^. We noted that the production of acetate in the MEA electrolyzer is higher than that in the three-compartment flow cell. In addition, a small amount of propionate was also detected after CO electrolysis (Supplementary Fig. 5). This can be attributed to (1) the reaction environment on the anion exchange membrane surface favors acetate formation and (2) the liquid products in MEA configuration are collected at anode side (see “Methods” for details), where a portion of the produced ethanol and n-propanol can be oxidized to acetate and propionate, respectively. These results are consistent with the previous literature^8,37,38^.

By normalizing the partial current densities of CORR with the electrochemically active surface area (ECSA) for all catalysts (Supplementary Fig. 6 and Supplementary Tables 2 and 3), we found that the specific activity of C_2+_ liquid product formations was improved in Cu(OD)_1__−__*x*_Ag_*x*_ (*x* = 0.1, 0.2, 0.3, 0.5) compared to Cu(OD), with optimal activity achieved in Cu(OD)_0.8_Ag_0.2_, while their specific activities of ethylene formation were similar (Supplementary Fig. 7a, b). C_2+_ products are generally believed to form via a common and early rate-determining step (RDS), either the coupling of two adsorbed CO molecules^39,40^ or the hydrogenation of CO^41^, the enhanced total C_2+_ product formation rates indicate that the addition of Ag accelerates the common RDS. The contrasting selectivity/rate trends of ethylene and C_2+_ liquid products suggest that the introduction of Ag likely changes the energetics of reaction steps leading to different groups of products after the RDS (see below).

### In situ surface-enhanced infrared absorption spectroscopic investigations

**Fig. 2:**
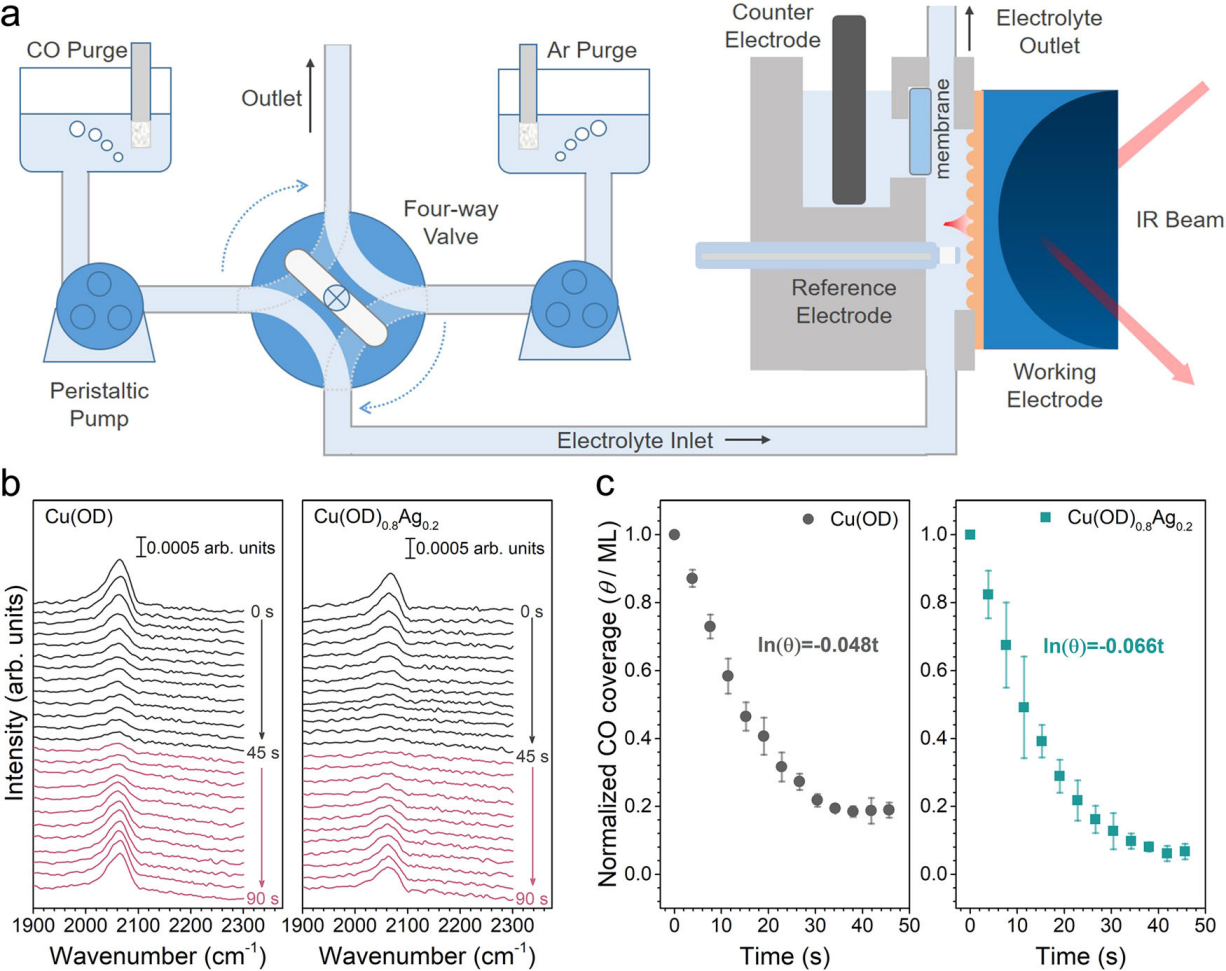
Desorption rate of CO_ad_ measured by surface-enhanced infrared absorption spectroscopy. **a** Schematic of the custom-designed two-compartment microfluidic flow electrochemical spectroscopic cell. **b** Representative time evolution of the infrared bands that result from CO bound to Cu surface recorded after removing CO in bulk solution by pulsing Ar-saturated electrolyte and subsequently delivering CO-saturated electrolyte at 45 s. **c** Normalized CO coverage obtained from the CO band in **b** as a function of time. The error bars represent the standard deviations from at least three independent measurements.

Weakly bound CO_ad_ on Cu(OD)_1__−__*x*_Ag_*x*_ samples was identified by measuring the CO_ad_ desorption rate with in situ surface-enhanced infrared absorption spectroscopy (SEIRAS). There is a general consensus that the linearly adsorbed CO is the key intermediate in the formation of C_2+_ products on Cu surface^42,43^, and the binding strength of CO_ad_ plays an important role in their reaction rates^39,44^. The binding strength of CO_ad_ on different catalysts can be compared by measuring the desorption rate of CO_ad_. In this work, we estimated the CO_ad_ desorption rate constant using a custom-designed two-compartment electrochemical spectroscopic cell capable of rapidly switching from a CO-saturated electrolyte to a CO-free electrolyte (Fig. 2a). The quick and complete electrolyte switch is essential for accurate CO_ad_ desorption measurements by minimizing the re-adsorption of CO molecules in the electrolyte. A control experiment shows that the H_2_O feed in the cathode compartment is completely replaced by D_2_O in less than 10 s (Supplementary Fig. 8).

To estimate the CO_ad_ desorption rate constants, we monitor the peak area of the linearly bound CO_ad_ at the vicinity of 2060 cm^−1^ in SEIRA spectra at −0.2 V_RHE_, at which no CORR is expected to occur^17,45^. CO-saturated 0.1 M KOH was first delivered to the cathode compartment. After the establishment of CO adsorption equilibrium, i.e., when the CO band intensity became time-independent, the electrolyte was switched to Ar-saturated 0.1 M KOH. The desorption of CO_ad_ was monitored by the decrease in the intensity of the corresponding band in SEIRAS. Representative spectra of Cu(OD) and Cu(OD)_0.8_Ag_0.2_ are shown in Fig. 2b. The areas of the CO band were integrated, normalized, and plotted with respect to time (Fig. 2c), in which the CO_ad_ desorption rate constant (K_D_) could be estimated by fitting with the first-order desorption rate expression of the form^46^

1 $${\theta }_{{{{{{\rm{CO}}}}}},{{t}}}={\theta }_{{{{{{\rm{CO}}}}}},0}{{{{{\rm{exp }}}}}}(-{{{{{\rm{K}}}}}}_{{{{{\rm{D}}}}}}{{t}})$$

where K_D_ is the desorption rate constant of CO_ad_, and *θ*_CO_,_*t*_ and *θ*_CO_,_0_ represent the CO coverage at any time and at *t* = 0, respectively. Since there inevitably are sites with varying CO binding strengths on the surface, the measured K_D_ represents a weighted average of all sites present on the surface. K_D_ is determined by the initial slope of ln(*θ*_CO_,_*t*_) (Supplementary Fig. 9), which is expected to be heavily influenced by weakly adsorbing sites. Although re-adsorption of CO is minimized by the demonstrated fast electrolyte switch, it cannot be completely eliminated, and thus the measured K_D_ is a lower bound of the desorption rate constant. This fact does not prevent the identification of sites of different CO adsorption energies among samples as long as the difference in the measured K_D_ values is outside the experimental errors. The K_D_ values were calculated to be 0.048 ± 0.004 s^−1^ on Cu(OD) and 0.066 ± 0.002 s^−1^ on Cu(OD)_0.8_Ag_0.2_, respectively, indicating a ~38% faster CO_ad_ desorption rate on Cu(OD)_0.8_Ag_0.2_ in comparison to that on Cu(OD) (Fig. 2c and Supplementary Fig. 9). This substantial difference suggests that the introduction of Ag in our sample decreases the average binding strength of CO likely by introducing weak binding sites. Further addition of Ag beyond 20% leads to lower K_D_ values, e.g., 0.051 ± 0.003 s^−1^ for Cu(OD)_0.5_Ag_0.5_ (Supplementary Figs. 9 and 10), which can be attributed to the agglomeration of Ag as revealed by structural characterization results that reduce its contact to the Cu(OD) surface (see below). The estimated K_D_ value decreases in the same order as the decrease of C_2+_ liquid products/ethylene ratio (i.e., Cu(OD)_0.8_Ag_0.2_ > Cu(OD)_0.5_Ag_0.5_ > Cu(OD)) (Fig. 1b). These results indicate that the increased proportion of weak CO binding sites is strongly correlated with the enhanced formation of C_2+_ liquid products on Cu(OD)_1__−__*x*_Ag_*x*_. The CORR performance of all catalysts in 0.1 M KOH at −0.7 V was evaluated and the FE_C2+ liquid products_/FE_ethylene_ ratio was observed to follow a similar trend on different catalysts compared with the results obtained in 1.0 M KOH (Fig. 1 and Supplementary Fig. 11). Thus, the concentration of OH^−^ of the electrolyte is unlikely to impact the underlying physicochemical origin for the high C_2+_ liquid products selectivity of our catalysts. This is consistent with our previous reports showing that electrolyte pH does not impact the C_2+_ product formations in the CORR^47,48^. We note the mass transport in the SEIRAS cell is different from that in the flow cell. However, the goal of our SEIRAS is to compare the average CO binding strength on different catalysts, which is a thermodynamic property of catalysts independent of mass transport, via estimation of their CO desorption rates at identical experimental conditions.

### Electrolysis in the switching CO/Ar atmosphere on Cu(OD)_0.8_Ag_0.2_

**Fig. 3:**
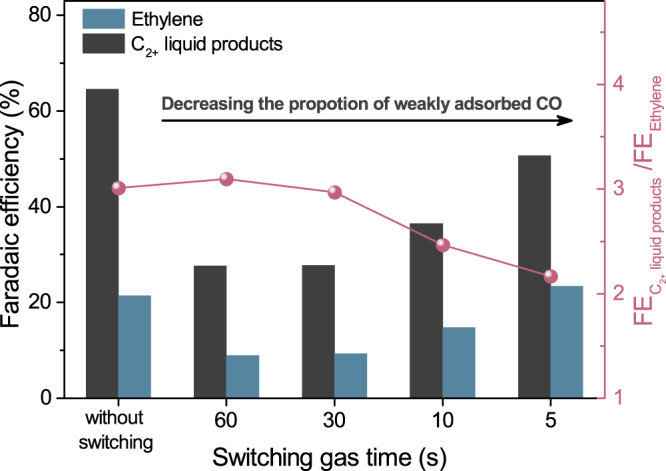
Electrolysis in the switching CO/Ar atmosphere on Cu(OD)_0.8_Ag_0.2_. Faradaic efficiency of multi-carbon liquid products and ethylene. The red circles show the corresponding Faradaic efficiency ratio of multi-carbon liquid products to ethylene. Constant current electrolysis at 100 mA cm^−2^ is conducted on Cu(OD)_0.8_Ag_0.2_ with switching inlet gas between CO and Ar at different frequencies (switch CO/Ar every 60, 30, 10, 5, and 0 seconds).

To further probe the potential role of weakly bound CO_ad_ in promoting C_2+_ liquid product formations over Cu(OD)_0.8_Ag_0.2_, we conducted electrolysis experiments with inlet gas periodically switching between CO and Ar at different frequencies (Fig. 3). The experiment setup is designed to avoid fluctuation in the cathodic gas flow (See “Methods” and Supplementary Fig. 12). By switching the gas feed from CO to Ar, the weakly bound CO_ad_ on the electrode surface is expected to desorb faster than that on the more strongly bound CO_ad_, while in switching the gas feed from Ar to CO, the dissolved CO in the electrolyte tends to adsorb more slowly on the weak binding sites than on the stronger binding sites^49,50^. Therefore, the proportion of CO_ad_ on weak binding sites can be effectively decreased by increasing the CO/Ar switch frequencies. The C_2+_ liquid products/ethylene ratio is significantly smaller at the CO/Ar switching interval of 5 s (Fig. 3), suggesting that CO adsorbed on weak binding sites is strongly correlated with the formation of C_2+_ liquid products. This ratio increases at longer switching intervals, and largely recovers to that in electrolysis with pure CO at the switching interval of 60 s. It indicates that the duration with substantial variations in the relative proportions of CO adsorbed on strong and weak binding sites due to electrolyte switching is much shorter than 60 s, and the product distribution is dominated by steady-state electrolysis. A decrease in the Faradaic efficiency for both C_2+_ liquid products and ethylene was observed in electrolysis with switching CO/Ar and was more significant at longer switching intervals. This can be attributed to the more promoted HER on the electrode surface due to more thoroughly desorbed CO_ad_ opening more available sites for HER, while CO_ad_ is less likely to be completely consumed or desorbed at shorter switching times.

### Structural characterizations

**Fig. 4:**
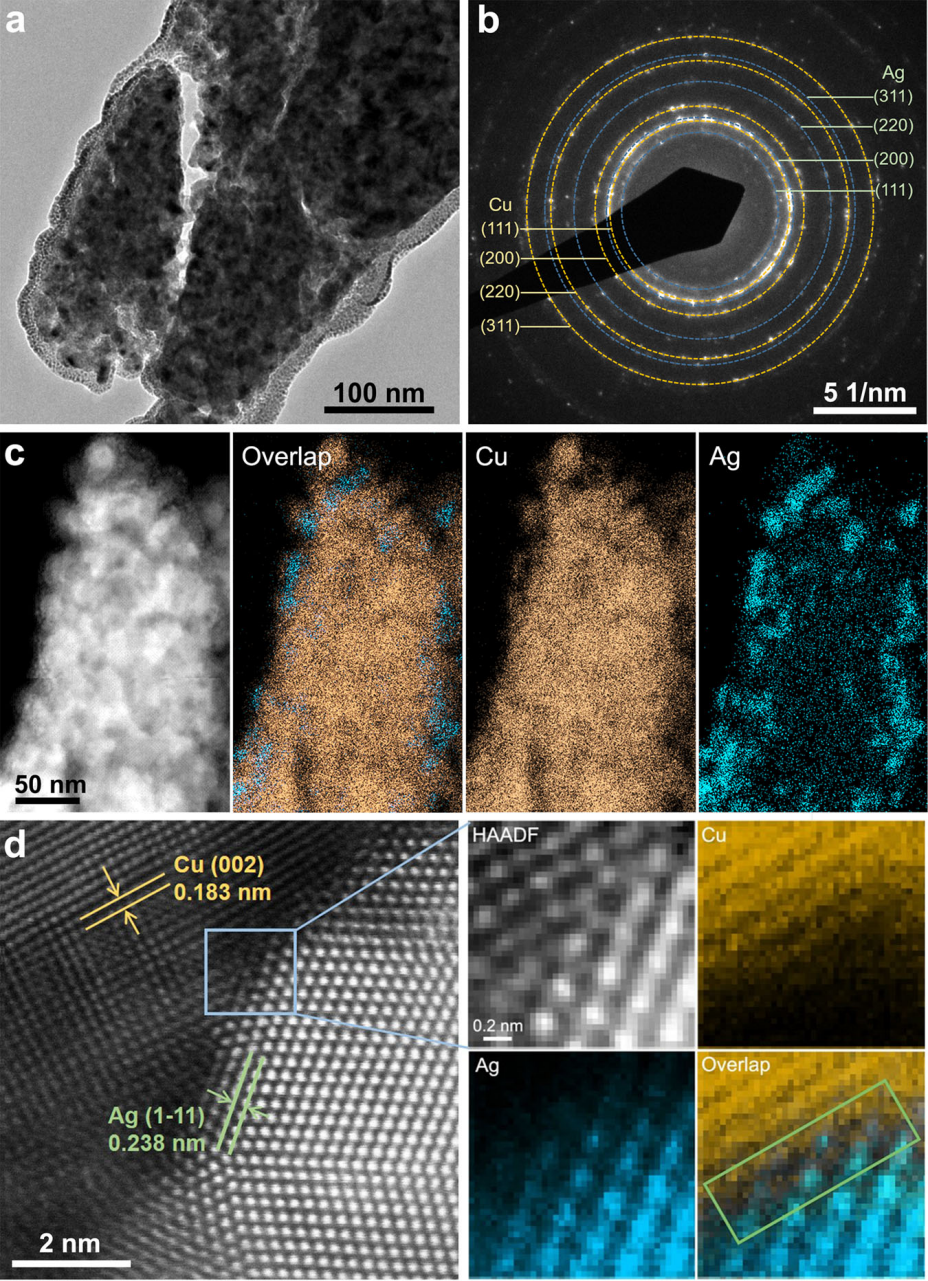
Structural characterization of Cu(OD)_0.8_Ag_0.2_ catalyst after CO reduction reaction. **a** TEM image of Cu(OD)_0.8_Ag_0.2_ catalyst cross-section cut by FIB. **b** Electron diffraction pattern of Cu(OD)_0.8_Ag_0.2_ catalyst shown in **a**. **c** HAADF-STEM image and corresponding EDS mapping of Cu and Ag. **d** HAADF-STEM image and the corresponding atomically resolved EDS mapping of Cu and Ag at the selected interface area. The atomic ratio between Cu and Ag at the interface (i.e., the area within the green box) was determined to be 1.12:1.

To investigate the origins of the weakly bound CO_ad_, we conducted transmission electron microscopy (TEM) characterizations on the post-electrolysis Cu(OD)_0.8_Ag_0.2_ sample prepared with the focused ion beam (FIB) technique. No significant morphology change was observed on the post-electrolysis Cu(OD)_0.8_Ag_0.2_ which remained to be plate-like (Supplementary Fig. 13). An ultra-thin specimen of its cross-section was cut out using FIB and transferred for TEM characterizations. TEM image shows that the post-electrolysis Cu(OD)_0.8_Ag_0.2_ is composed of small grains with sizes of ~10–20 nm (Fig. 4a). Electron diffraction (ED) patterns revealed its polycrystalline nature with the diffraction spots assigned to face-centered cubic (fcc) Cu and fcc Ag (Fig. 4b). These results are consistent with powder X-ray diffraction (XRD) characterizations of post-electrolysis Cu(OD)_0.8_Ag_0.2_ (Supplementary Fig. 14). No metal oxide phases, e.g., CuO or Cu_2_O, were identified, suggesting that the CuO was reduced to metallic Cu after CO electrolysis. The distribution of Ag and Cu in this microstructure was examined using the high-angle annular dark-field scanning transmission electron microscopy (HAADF-STEM) combined with energy dispersive X-ray spectroscopy (EDS) mapping (Fig. 4c). Cu is quite uniformly distributed along the cross-section sample, while Ag mainly exits on the outer layers of Cu. High-resolution transmission electron microscopy (HRTEM) images revealed the presence of phase boundaries at the interface between Ag and Cu particles. HAADF-STEM image in Fig. 4d shows a well-defined interface between Cu and Ag. The atomic structures correspond to the metallic Cu and Ag with crystallographic zone axis of [100] and [110], respectively. The orientation relationship is confirmed as Cu(022)//Ag(1–11) and Cu[100]//Ag[110]. Other orientations, such as fcc Ag (200) planes and fcc Cu (200) planes, fcc Ag (200) planes and fcc Cu (111) planes, are also observed to form interfaces as shown in Supplementary Fig. 15. To investigate the composition of Cu–Ag interface, we performed the atomically resolved EDS mapping on the interface area using the K edge of Cu and the L edge of Ag, respectively (Fig. 4d). The interface was confirmed to form between metallic Cu and Ag with an atomic ratio of ~1:1. No Ag–Cu alloy was identified, which was expected because Ag and Cu are highly immiscible spanning a broad composition indicated by their binary phase diagram^51^. Post-reaction XPS characterizations on all Cu(OD)_1__−__*x*_Ag_*x*_ electrodes showed a similar distribution of Cu(0) and Cu(I) (Supplementary Fig. 16), in which Cu(I) was most likely due to the inevitable sample exposure to the air^52^, suggesting that the valence state of Cu was not noticeably impacted by the addition of Ag. Thus, we consider that the induced surface Cu–Ag phase boundaries during CORR are largely responsible for the presence of weakly bound CO_ad_ by providing adsorption sites with weaker CO binding strength.

### Effect of ball milling time on C_2+_ liquid products formation

**Fig. 5:**
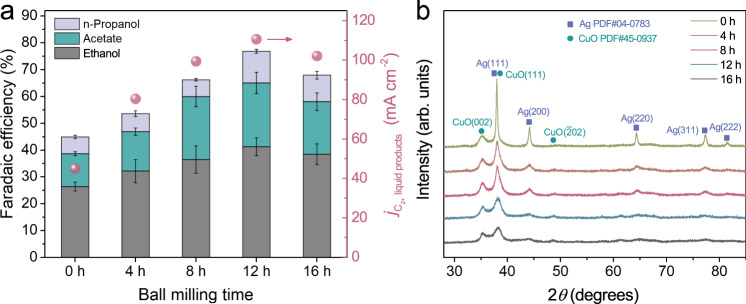
Ball milling time-dependent CORR performance and XRD patterns. **a** Faradaic efficiency of multi-carbon liquid products. The red circles show the corresponding partial current densities of C_2+_ liquid products. **b** XRD patterns of as-synthesized catalyst powder before electrolysis.

The ball milling time shows a significant impact on the C_2+_ liquid products formation. Figure 5a shows the Faradaic efficiencies and partial current densities of C_2+_ liquid product formations of Cu(OD)_0.8_Ag_0.2_ samples with different milling times (i.e., 0 h, 4 h, 8 h, 12 h, and 16 h) at −0.56 ± 0.01 V_RHE_. The 0 h sample exhibits similar selectivity and activity for C_2+_ liquid product formations with Cu(OD) (Fig. 5a), suggesting that a simple mixture of CuO and Ag powders was not able to provide the weak adsorption sites for CO. This is also supported by the SEIRAS results that the non-ball-milled CuO and Ag powder catalyst (Cu:Ag = 0.8:0.2) exhibits a similar K_D_ value of 0.047 ± 0.001 s^−1^ compared with Cu(OD) catalyst (Supplementary Fig. 17). As the milling time increases from 0 to 12 h, substantial increases in both Faradaic efficiencies and partial current densities were observed. A prolonged milling time of 16 h leads to a slight decrease in C_2+_ liquid product formations, which can be attributed to the zirconia contamination from the grinding media demonstrated in XPS examinations (Supplementary Fig. 18). The XRD patterns only show the diffraction features of monoclinic CuO lattice and fcc Ag lattice, respectively (Fig. 5b). No alloy peaks can be detected. With increasing ball milling time, the diffraction peaks of Ag decrease in intensity and become broadened in width, indicating a significant reduction in its crystallite size. No obvious change in CuO peaks is observed as its preparation already involves a 12-h milling process (see “Methods” for details). The homogeneity of the CuO and Ag mixture improves with extended ball milling time (Supplementary Fig. 19a–c). The smaller-sized Ag crystallites as well as their more homogeneous distribution could be beneficial for the formation of the Cu–Ag phase boundary during CO electrolysis. This is also supported by the measured K_D_ of 0.054 ± 0.002 s^−1^ for the 4-h-milled Cu(OD)_0.8_Ag_0.2_ (Supplementary Fig. 20), indicating its weak CO adsorption sites are more than those of Cu(OD) but less abundant than those of 12-h-milled Cu(OD)_0.8_Ag_0.2_. In addition to ball milling time, the Ag content is found to impact the homogeneity of the CuO and Ag mixture. As increasing the Ag:Cu ratio to 0.5:0.5, the 12-h-milled Cu(OD)_0.5_Ag_0.5_ shows reduced homogeneity with Ag agglomeration (Supplementary Fig. 19d), which is likely to limit the formation of Cu–Ag phase boundaries and thus leading to lower K_D_ values (Supplementary Fig. 9).

## Discussion

The introduction of Ag to Cu via ball milling creates weak CO binding sites that are distinct from sites on the Cu surface, which is likely the key to the enhanced rates and selectivities for C_2+_ liquid products in the CORR. Based on the TEM imaging and elemental mapping of the Cu(OD)_*x*_Ag_1__−__*x*_ catalysts, it is clear that the interaction between the two elements is mainly at the interface between the Cu and the Ag domains (Fig. 4), rather than through alloying. This is expected as Cu and Ag tend to phase segregate rather than form an alloy^34,53^. In addition, our recent collaborative work showed that the alloying of Cu and Ag is unlikely to change the CO binding strength as the peak position of linearly adsorbed CO band on a homogeneous Ag–Cu alloy remained the same as that of bare Cu^22^. In contrast, the adsorbed CO band of Cu(OD)_0.8_Ag_0.2_ exhibits a clear shift to higher wave numbers compared to Cu(OD), indicating weaker CO adsorption (Supplementary Fig. 21). The weak CO adsorption sites are unlikely to be composed of Ag atoms because the SEIRAS results show that CO does not adsorb on bare Ag surface (blue trace in Supplementary Fig. 21). This is expected as the CO binding strength is significantly lower on Ag than on Cu^30,54^. Thus, it is reasonable to deduce that two types of Cu sites are present on Cu(OD)_1__−__*x*_Ag_*x*_ catalysts, i.e., native Cu(OD) sites, and weak CO adsorption sites consist of Cu atoms located at the interface of immiscible Cu and Ag particles, whose electronic structure is likely modified by the neighboring Ag atoms. The weak CO binding sites identified by in situ SEIRAS on the Cu(OD)_1__−__*x*_Ag_*x*_, as compared to Cu(OD), can be assigned to the Cu–Ag interfacial sites. Ag is well known to be a weak CO binding metal, so the fact that Cu–Ag interfacial sites bind less strongly than native Cu(OD) sites should not come as a surprise. Further, the Cu and Ag domains in Cu(OD)_1__−__*x*_Ag_*x*_ catalysts are tens of nanometers in size, suggesting that the interfacial area is relatively minor as compared to that of Cu domains. This explains the relative constant specific ethylene production rate upon introducing Ag to Cu(OD), as the CORR activity on Cu domains remains largely unchanged (Supplementary Fig. 7b). It follows that the enhanced rates and selectivities for C_2+_ liquid products are correlated with the Cu–Ag interfacial sites with relative weak CO adsorption energies on all samples with different element ratio and ball milling time.

Enhanced formation of C_2+_ liquid products on Cu(OD)_1__−__*x*_Ag_*x*_ could be attributed to the lower C and OH affinities of the Cu–Ag interfacial sites, which have been predicted to favor oxygenates over ethylene^30,31^. The RDS for C_2+_ product formations on the Cu surface was suggested to be the CO_ad_ hydrogenation to COH_ad_ by our recent electrokinetic study^43^. The subsequent C–C coupling process was proposed to occur between COH_ad_ and CO_ad_ forming HOCCO_ad_^43^. Investigations based on theoretical calculation reported that HOCCO_ad_ was favored to go through a hydro-dehydroxylation path forming CHCO_ad_ with the hydrogenation of α-C^30,31^. The further hydrogenation of CHCO_ad_ was shown to form ethylene if either C atom is hydrogenated and to form oxygenates if the O is hydrogenated respectively^31^. While the C affinity of the surface could be used to predict the selectivity between oxygenates and ethylene, calculated surfaces favoring oxygenates tend to have both low C and O affinities. This is expected as the CO adsorption energy is reported to be linearly correlated with the OH binding energy on coinage metals^55^. It could reasonably be inferred that the Cu–Ag interfacial sites exhibit weaker affinity to both C and OH. Thus, our experimental results appear to agree well with this computational prediction. The significantly higher rates for oxygenates upon introduction of Cu–Ag interfacial sites suggest that these sites are exceptionally active because the interfacial sites account for a minor fraction of Cu sites due to the large particle size of both Cu and Ag. The ethylene rate remains largely unaffected with the introduction of Ag (Supplementary Fig. 7b). This indicates that the majority of Cu sites are not influenced by Ag, which is expected due to the lack of alloying of Cu and Ag. With our methodological development in the measurement of CO binding energy, this is the experimental verification of the computational prediction that the affinities of C and O are reliable descriptors for oxygenates/ethylene selectivity in the CORR. Our Cu(OD)_*x*_Ag_1__−__*x*_ catalysts exhibit a distinct working principle from other reported Cu–Ag catalysts typically prompting C–C coupling^32^ or introducing tandem process^56,57^. The exploration of other preparation methods, including galvanic replacement, ion-implementation, to form and exploit dense weak CO adsorption sites on Cu (e.g., small Cu particles on Ag with a large atomic ratio between Cu and Ag) can be fruitful strategies to further improve the rate and selectivity of CORR.

## Methods

### Materials

Cu powder (<45 μm, 99.7% trace metals basis), potassium hydroxide (semiconductor grade, 99.99% trace metals basis), and Nafion solution (5 wt%) were purchased from Sigma-Aldrich. Ag powder (0.7–1.3 μm, 99.9% trace metals basis) and IrO_2_ powder (99.99% trace metals basis) were purchased from Alfa Aesar. Carbon monoxide (99.999%) and argon (99.999%) were purchased from Air Liquide. The carbon fiber paper support (Sigracet 39 BC) was purchased from the Fuel Cell Store. The electrolyte solutions were prepared using Milli-Q water (18.2 MΩ cm).

### Synthesis

The Cu(OD)_1__−__*x*_Ag_*x*_ catalysts in this work were synthesized through a ball milling process. CuO powder was first prepared by heating Cu powder in a muffle furnace (Hefei Kejing Materials Technology Co., Ltd., KSL1100, China) in air at 500 °C for 2 h. Ten milligrams of this CuO powder was then added to two zirconia milling jars (35 ml) with 30 g of zirconia grinding balls (3 mm in diameter) and 8 ml of ethanol. The CuO powder was then milled for 12 h at 800 rpm in a planetary miller (Fritsch, PULVERISETTE 7). The milled CuO was collected and cleaned with Milli-Q water multiple times and was dried under vacuum at 50 °C for 6 h. To prepare Cu(OD)_1__−__*x*_Ag_*x*_ catalysts, the CuO powder was mixed with Ag powder with a desired atomic ratio and milled at identical conditions for different times.

### Electrode preparation

To prepare the gas-diffusion electrode for Cu(OD)_1__−__*x*_Ag_*x*_, an ink suspension was first prepared by mixing 100 mg as-synthesized material, followed by 5.0 ml isopropanol, and 200 µl Nafion solution by sonicating for 20 min. The ink solution was then airbrushed (using Ar as a carrier gas) onto the microporous layer of Sigracet 39 BC with a size of 5 × 6 cm^2^. All electrodes were prepared with the same Cu loading of 0.8 mg cm^−2^. After drying under vacuum to thoroughly remove the residual solvent, a 1 × 2.5 cm^2^ gas-diffusion electrode was cut and assembled into a custom-designed flow cell electrolyzer exposing a 1 cm^2^ electrode area for reactivity measurement.

### Electrocatalytic measurements in the three-compartment flow cell

CO electrolysis was performed in a three-channel microfluidic flow cell with a channel dimension of 2 cm × 0.5 cm × 0.2 cm. A piece of anion-conducting membrane (Selemion AMV AGC Inc.) was used to separate the cathode and anode chambers. The IrO_2_-coated carbon fiber paper with a mass loading of 1.0 mg cm^−2^ was used as the counter electrode, and a leak-free Ag/AgCl (3.4 M KCl, Innovative Instruments Inc.) was used as the reference electrode. The electrolyte was 1.0 M KOH with a pH of ~13.7. CO gas was delivered into the cathode chamber at a flow rate of 15.0 cm^3^ min^−1^ using a mass flow controller (MKS Instruments Inc.) and calibrated using Agilent ADM flow meter. The gas-phase backpressure in the microfluidic flow cell was regulated using a backpressure controller (Cole-Parmer). The flow rates of catholyte and anolyte were both set to be at 2 ml min^−1^ via a peristaltic pump (Cole-Parmer). The catalysts were pretreated by reducing at −5 mA cm^−2^ for 5 min before measurements. Chronopotentiometry experiments were conducted to evaluate the CO electroreduction performance using a Gamry Reference 600+ Potentiostat. The measured potential was manually IR corrected after the electrolysis and was converted to RHE in which E (vs RHE) = E (vs Ag/AgCl) + 0.210 V + 0.0591 V × pH.

Gas-phase products were quantified using a gas chromatograph (Agilent 7890B). Liquid products were quantified by a Bruker AVIII 400 MHz NMR spectrometer with water suppression using the excitation sculpting method. The NMR sample was prepared by mixing 500 µl of the electrolyte with 100 µl of D_2_O (Sigma-Aldrich, 99.9%) and 0.05 mM dimethyl sulfoxide (Alfa Aesar, ≥99.9%) as an internal standard.

In the gas switching experiments, two mass flow controllers continuously flow CO and Ar gas at a steady flow rate into a four-way valve (Supplementary Fig. 12). During the test, one of these two gas feeds was delivered into the flow cell while the other flows directly to the effluent by switching the four-way valve without altering the working status of mass flow controllers. In addition, a backpressure controller was connected to the gas outlet of the flow cell, followed by a flow meter. No change in pressure and flow rate was observed during the gas switch. The gas-phase products were collected in an airbag and sampled using a gastight syringe (Hamilton) for GC analysis.

### Electrocatalytic measurements in the MEA electrolyzer

The CO electrolysis in the MEA configuration was performed in a custom-designed two-compartment 100 cm^2^ (11.6 cm × 8.6 cm) MEA electrolyzer (see Supplementary Fig. 3) powered by a DC power supply (Z36-18-U, TDK-Lambda). An anion exchange membrane (Fumasep FAA-3-PK-13) was positioned between the cathode and the IrO_2_-coated porous titanium electrode (anode). The anode was prepared using a method that has been reported^58^. Using a mass flow controller, 60 sccm of humidified CO was fed into the cathode flow channels, while the anode side was fed with 1.0 M KOH electrolyte at a flow rate of 50 ml min^−1^ using a peristaltic pump. A custom-designed cold trap was used for separating liquid products and gas products in the outlet flow from the cathode side. For reactivity data in MEA configuration, acetate was assumed to be a 4e^−^ reduction product^37^.

### Electrochemically active surface area measurements

The ECSAs of the Cu(OD)_1__−__*x*_Ag_*x*_ electrodes were determined by measuring the electrochemical double-layer capacitance (C_DL_) in an H-cell^17,18^. All electrodes were electrochemically reduced at −5 mA cm^−2^ for 5 min before ECSA measurements. Cyclic voltammetry was performed on each electrode at various scan rates (i.e., 20, 40, 60, 80, and 100 mV s^−1^) at an Ar atmosphere in 0.1 M HClO_4_ (Supplementary Fig. 6). The potential region of no Faradaic current ranged from −300 to −150 mV vs Ag/AgCl (3 M KCl). The observed current was plotted versus the scan rate to obtain the capacitance of each electrode. Considering that the total C_DL_ was contributed by the combination of Cu and Ag, the ECSA of each electrode was calculated from the formula ${{{{{\rm{ECSA}}}}}}={C}_{{{{{\rm{CuAg}}}}}}\times (\frac{{Cu}\%}{{C}_{{{{{\rm{Cu}}}}}}\; {{{{{\rm{foil}}}}}}}+\frac{{Ag}\%}{{C}_{{{{{\rm{Ag}}}}}}\; {{{{{\rm{foil}}}}}}})$, where *C*_CuAg_, *C*_Cu foil_ and *C*_Ag foil_ represent the capacitance of Cu(OD)_1__−__*x*_Ag_*x*_ catalyst, Cu foil, and Ag foil, respectively. Cu% and Ag%, which represent the atomic ratio of Cu and Ag on the surface, were obtained from XPS peak fitting. However, the partial current densities of multi-carbon liquid products and ethylene were normalized only by the ECSA of Cu for each catalyst because Ag is believed to be the CORR inactive catalyst. The ECSA of Cu for each catalyst was calculated from the formula ${{{{{{\rm{ECSA}}}}}}}_{{{{{\rm{Cu}}}}}}={C}_{{{{{\rm{CuAg}}}}}}\times \frac{{Cu}\%}{{C}_{{{{{\rm{Cu}}}}}}{{{{{\rm{foil}}}}}}}$. The double-layer capacitive current density of the bare carbon support measured at identical conditions is two orders of magnitude lower than that of the carbon paper-supported Cu electrodes (Supplementary Fig. 22). Therefore, the electrochemical response from carbon support was not considered in this work.

### Physical characterization

The powder X-ray diffraction patterns were recorded on an automated powder X-ray diffractometer (40 kV, 40 mA, Bruker, Model D8Advance) using a Cu Kα radiation source (l = 1.54178 Å). The field-emission scanning electron microscope images were recorded on a Merlin FESEM from Zeiss. SEM-EDS elemental maps were acquired with TESCAN VEGA3 SEM equipped with an Oxford Instruments x-act detector. X-ray photoelectron spectroscopy measurements were carried out using a PHI Quantera II. The resulting spectra were analyzed using the CasaXPS software package (Casa Software Ltd., U.K.). TEM sample was prepared using Ga^+^ focused ion on a ZEISS AURIGA® Field Emission-SEM implemented with CrossBeam® Workstations. TEM images and STEM-EDS elemental maps were acquired with a JEOL JEM-F200 TEM.

### Desorption rate measurement by in situ SEIRAS

The Cu(OD) and the Cu(OD)_1__−__*x*_Ag_*x*_ electrodes for SEIRAS investigation were prepared on Au film that was predeposited onto a silicon ATR crystal by chemical deposition. In specific, the silicon prism was mechanically polished using a 0.05 µm Al_2_O_3_ slurry and sonicated in acetone and water to remove any residue Al_2_O_3_ particles. After polishing, the silicon prism was immersed in a 3:1 by volume solution of H_2_SO_4_ (Sigma-Aldrich, 95–98%) and H_2_O_2_ (Sigma-Aldrich, 30%) for 20 min to remove possible organic contaminants on the prism. Following that, the reflecting plane of the prism was immersed in NH_4_F (Sigma-Aldrich, 40%) for 90–120 s to remove the surface oxides as well as create a hydrogen-terminated surface for improving the adhesion of the Au film. The Au film was then chemically deposited by immersing the reflecting surface in a 4.4:1 by volume mixture of 2% HF and Au plating solution consisting of 5.75 mM NaAuCl_4_·2H_2_O, 0.025 M NH_4_Cl, 0.075 M Na_2_SO_3_, 0.025 M Na_2_S_2_O_3_·5H_2_O, and 0.026 M NaOH for 4 min^59^. The electrode was prepared by dropping the catalyst ink suspension onto the Au film. A custom-designed two-compartment, three-electrode spectroelectrochemical cell in flow configuration was employed for the in situ SEIRAS test. A peristaltic pump (Cole-Parmer) was employed to enable the quick switch among different electrolytes in this cell design. The cell was assembled on a Bruker INVENIO-S FTIR spectrometer equipped with a liquid nitrogen-cooled MCT detector and connected to a BioLogic SP-150e potentiostat. All spectra were collected at a 4 cm^−1^ spectral resolution and were presented in absorbance units. In a typical process, the catalyst-deposited Si prism was used as the working electrode with a graphite rod as the counter electrode and saturated Ag/AgCl as the reference electrode. Before collecting the spectra, all electrodes were electrochemically reduced at −5 mA cm^−2^ for 5 min and the background was then taken at 0.2 V_RHE_ in Ar-saturated 0.1 M KOH (pH 12.8).

## Supplementary information

Supplementary Information in PDF

## Data Availability

All relevant data are available from the corresponding authors upon reasonable request.
