# Supplementary material for: Weak CO binding sites induced by Cu–Ag interfaces promote CO electroreduction to multi-carbon liquid products
Source: Nat Commun. 2023 Feb 8;14:698. doi: 10.1038/s41467-023-36411-5 (PMC9908878; doi:10.1038/s41467-023-36411-5)
Supplement: Supplementary file 1 — Supplementary Information in PDF [file 41467_2023_36411_MOESM1_ESM.pdf]

Supplementary Information for

Weak CO Binding Sites Induced by Cu-Ag Interfaces Promotes CO Electroreduction to Multi-Carbon Liquid Products

Jing Li[1,4], Haocheng Xiong[1,2,4], Xiaozhi Liu[3], Donghuan Wu[1], Dong Su[3], Bingjun Xu[2] & Qi Lu[1]

[1]State Key Laboratory of Chemical Engineering, Department of Chemical Engineering, Tsinghua University, Beijing 100084, China

[2]College of Chemistry and Molecular Engineering, Peking University, Beijing, 100871, China

[3]Beijing National Laboratory for Condensed Matter Physics, Institute of Physics, Chinese Academy of Sciences, 100190, China

[4]These authors contributed equally: Jing Li, Haocheng Xiong.

Email: luqicheme@mail.tsinghua.edu.cn

Table of Contents:

Supplementary Figures 1-22

Supplementary Tables 1-3

Supplementary References

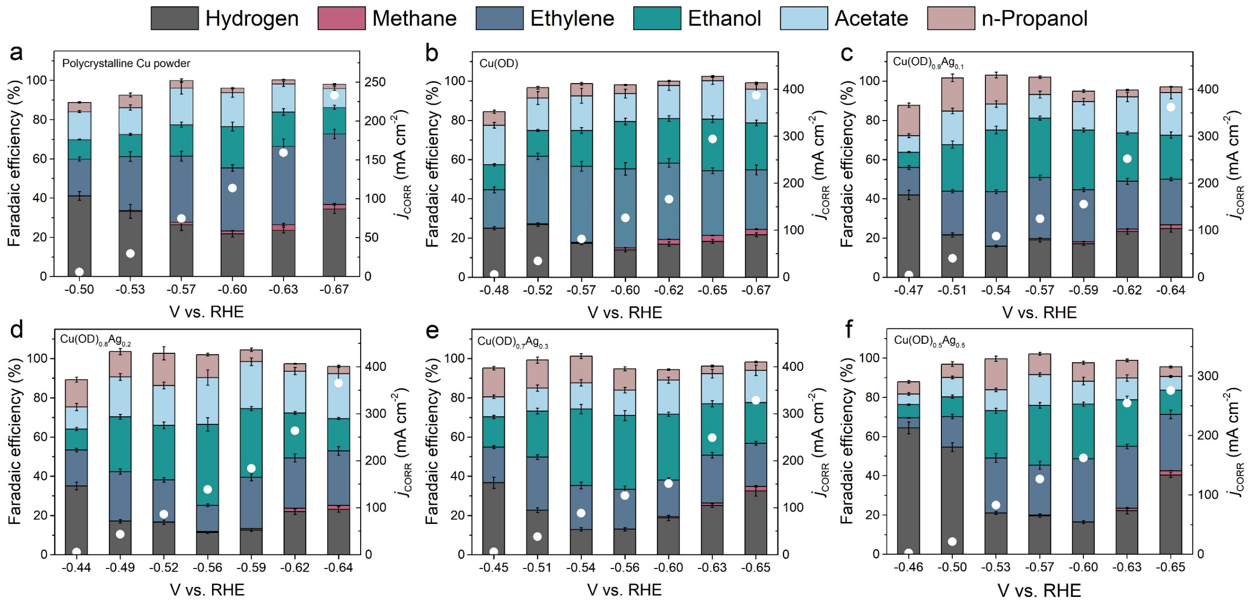

Supplementary Figure 1. CO electroreduction performance of different catalysts. Total current density and Faradaic efficiency of CO electroreduction on (a) polycrystalline Cu powder, (b) Cu(OD), (c) $Cu(OD)_{0.9}Ag_{0.1}$, (d) $Cu(OD)_{0.8}Ag_{0.2}$, (e) $Cu(OD)_{0.7}Ag_{0.3}$ and (f) $Cu(OD)_{0.5}Ag_{0.5}$. The error bars represent the standard deviation from at least three independent measurements.

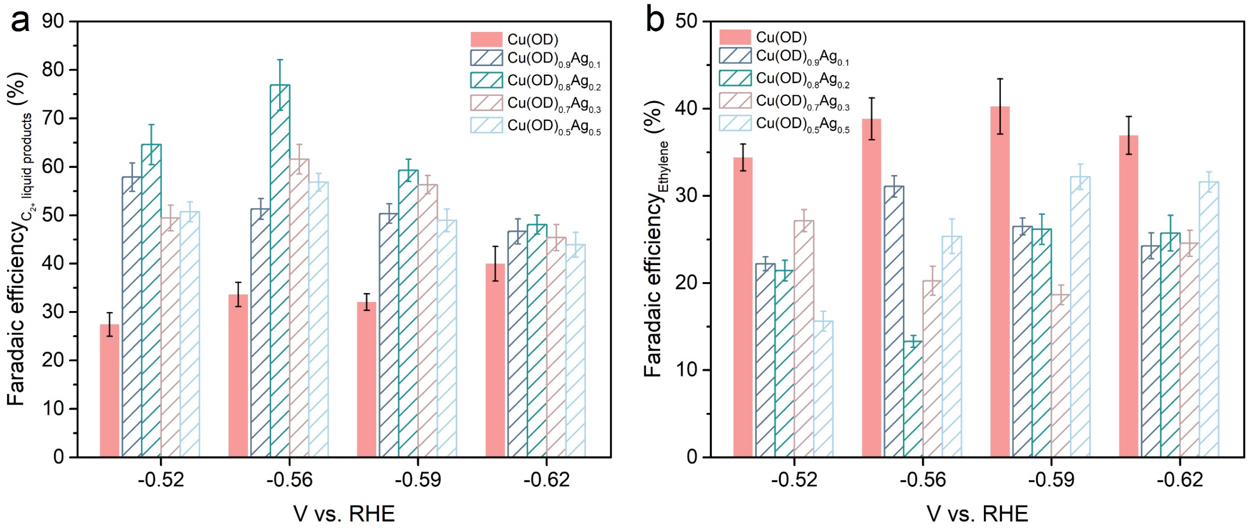

Supplementary Figure 2. Comparison of Faradaic efficiencies of C_{2+} liquid products and ethylene on different catalysts. Faradaic efficiencies of (a) C_{2+} liquid products and (b) ethylene on different catalysts at various applied potentials (-0.52±0.01 V, -0.56±0.01 V, -0.59±0.01 V, -0.62±0.01 V) in 1 M KOH. The error bars represent the standard deviation from at least three independent measurements.

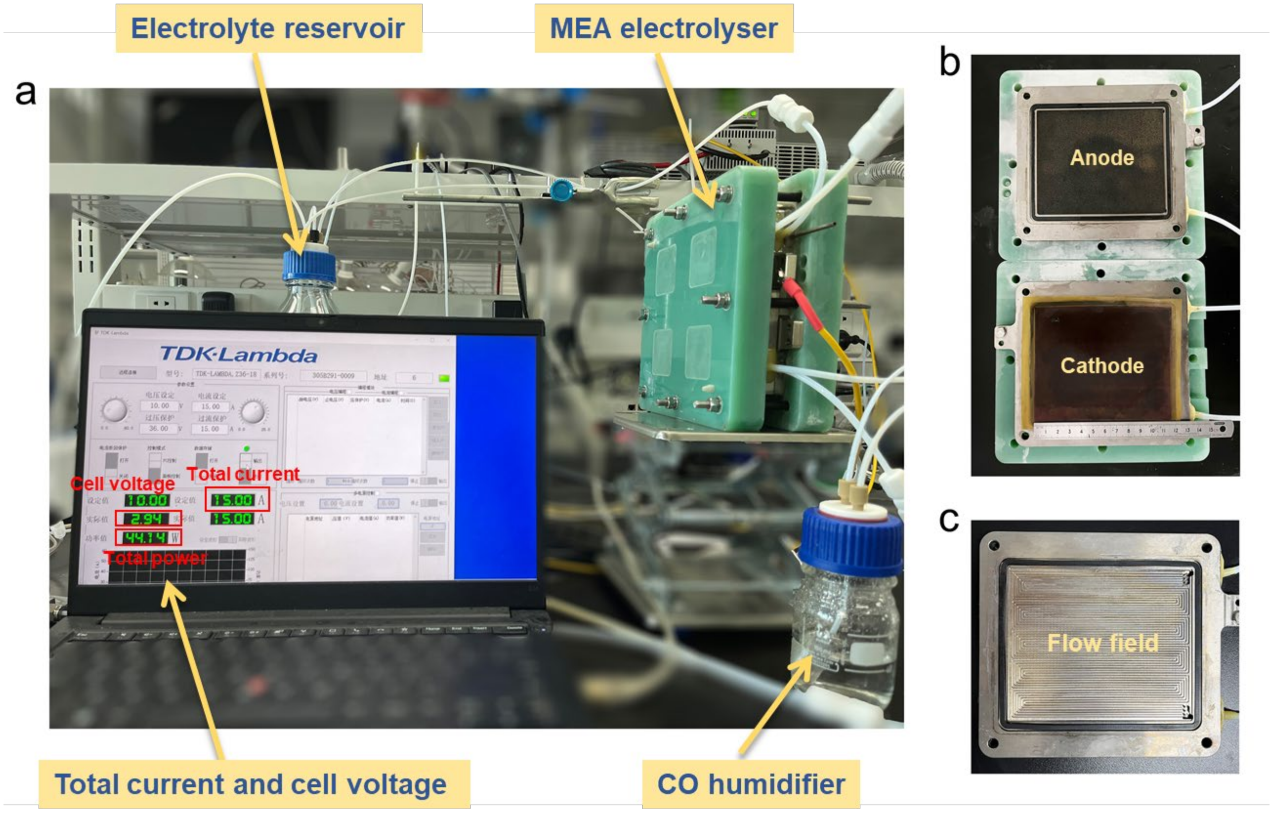

Supplementary Figure 3. Images of our custom-designed 100 cm^2 MEA system for CO electrolysis. (a) MEA electrolyser under CO reduction operation at a total current of 15 A using $Cu(OD)_{0.8}Ag_{0.2}$ as catalyst. (b) Anode and cathode electrodes after 103-h CO electrolysis. c End plate current collector with our custom-designed flow field.

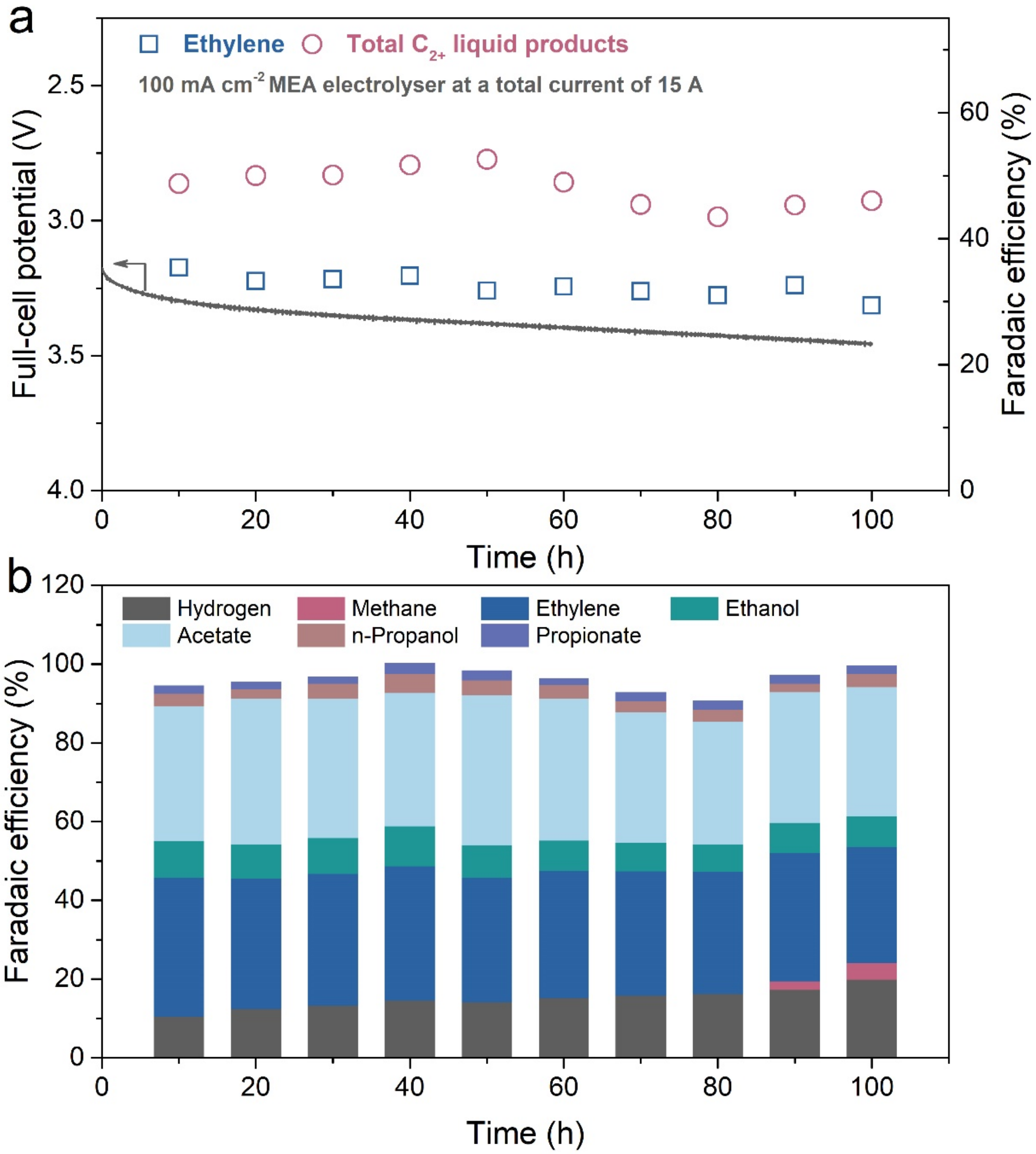

Supplementary Figure 4. CORR performance of the Cu(OD) catalyst in the 100 cm^{-2} MEA electrolyser. a Full-cell voltage and **b** product distribution with respect to time at a constant current of 15 A.

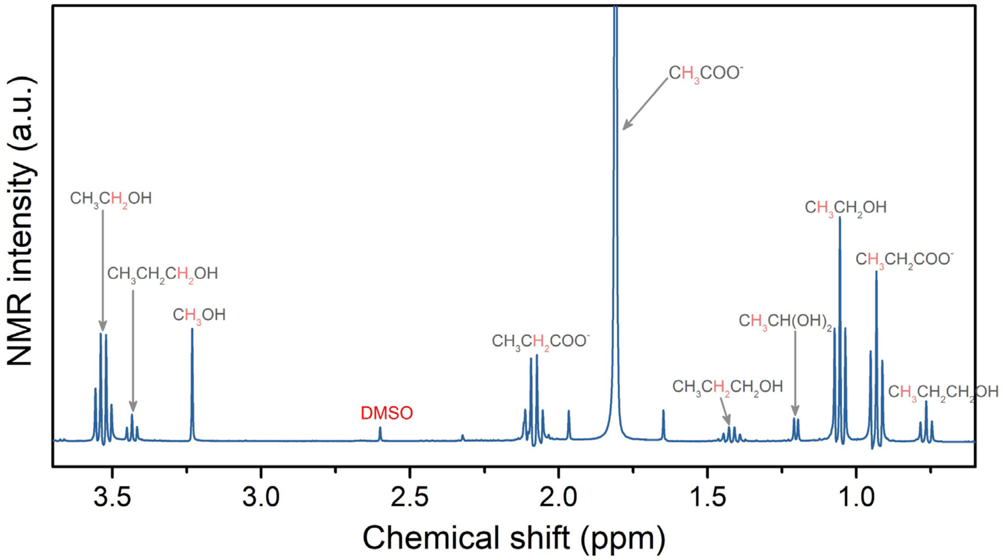

Supplementary Figure 5. Representative ^{1}H NMR spectrum of the electrolyte sampled in CORR of the $Cu(OD)_{0.8}Ag_{0.2}$ catalyst in the 100 cm^{-2} MEA electrolyser. The Faradaic efficiencies of other minor liquid products such as CH_3OH and $CH_3CH(OH)_2$ are less than 2% and thus are not discussed in this work.

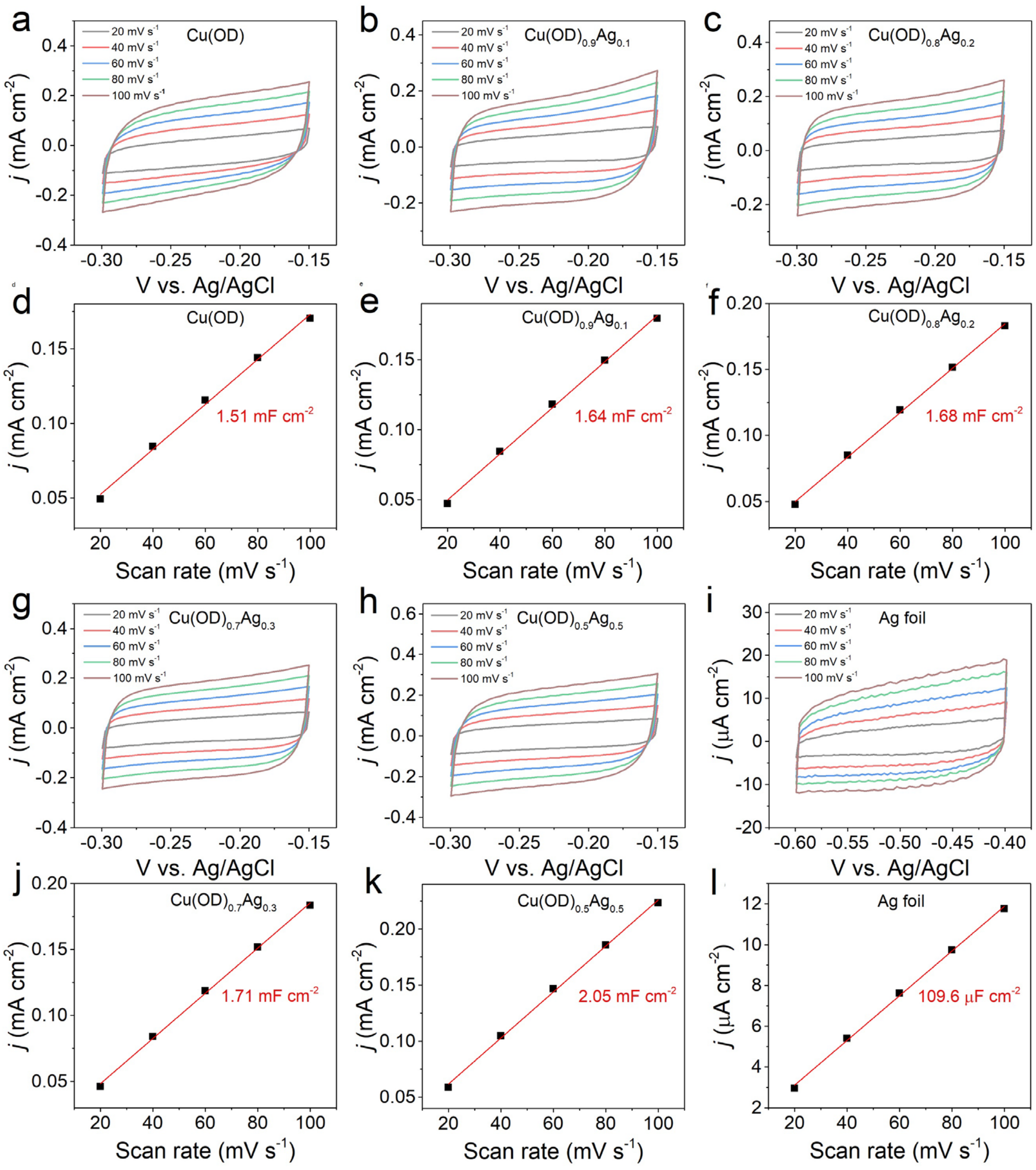

Supplementary Figure 6. ECSA measurements for different electrodes. Cyclic voltammetry study of (a) Cu(OD), (b) $Cu(OD)_{0.9}Ag_{0.1}$, (c) $Cu(OD)_{0.8}Ag_{0.2}$, (g) $Cu(OD)_{0.7}Ag_{0.3}$ and (h) $Cu(OD)_{0.5}Ag_{0.5}$, and (i) Ag foil. (d-f, j-l) Measured double layer charging current vs. scan rate for corresponding electrodes. Calculated roughness factors of different electrodes are shown in Supplementary Table 2.

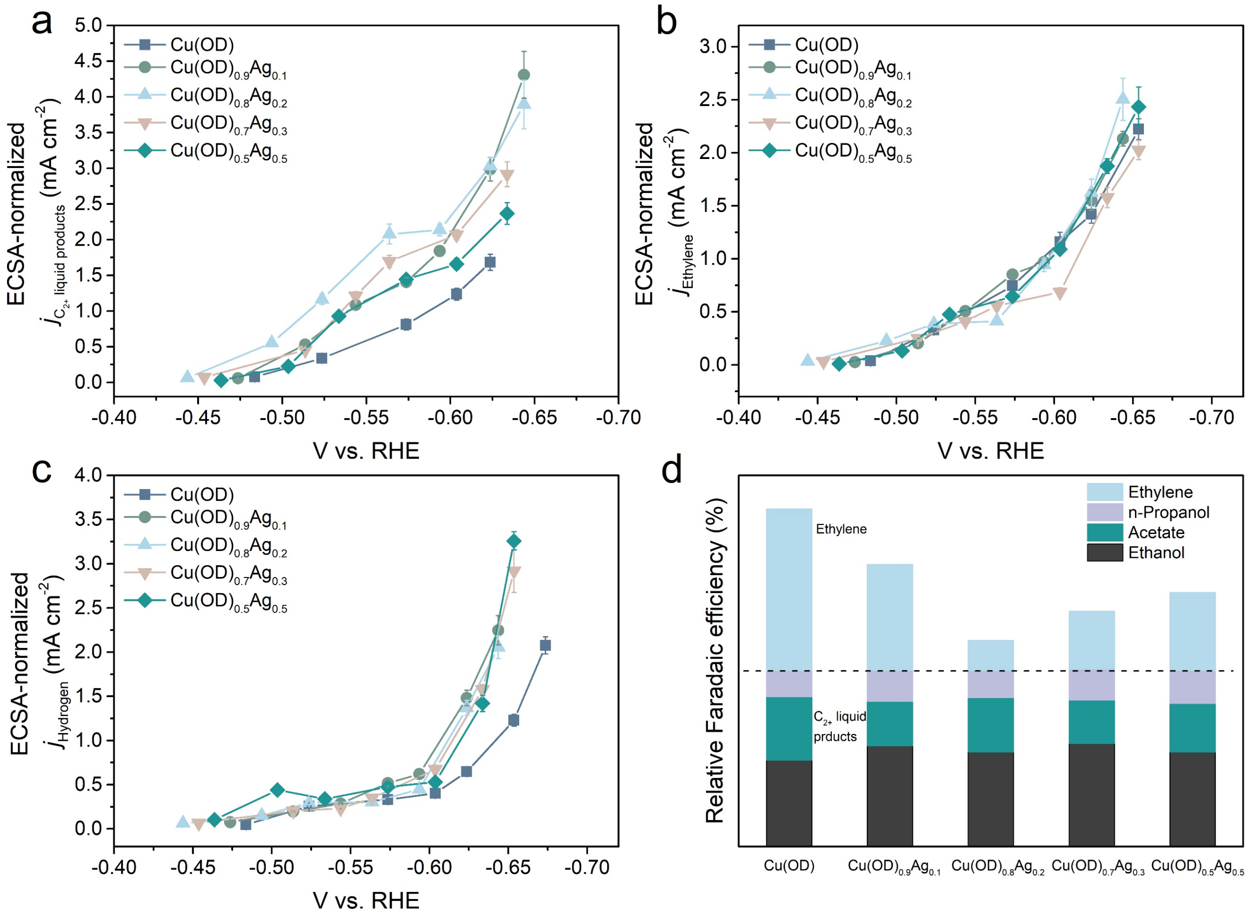

Supplementary Figure 7. ECSA-normalized partial current densities of different products and relative Faradaic efficiency between C_{2+} liquid products and ethylene. ECSA-normalized partial current densities of (a) C_{2+} liquid products, (b) ethylene, and (c) hydrogen for different electrodes. (d) Relative Faradaic efficiency between C_{2+} liquid products and ethylene at –0.56±0.01 V. Below the dashed line: Faradaic efficiency of ethanol, acetate and n-propanol for each electrode renormalized after omitting the Faradaic efficiencies of hydrogen and ethylene. Above the dashed line: Faradaic efficiency of ethylene relative to those of C_{2+} liquid products. The error bars represent the standard deviation from at least three independent measurements.

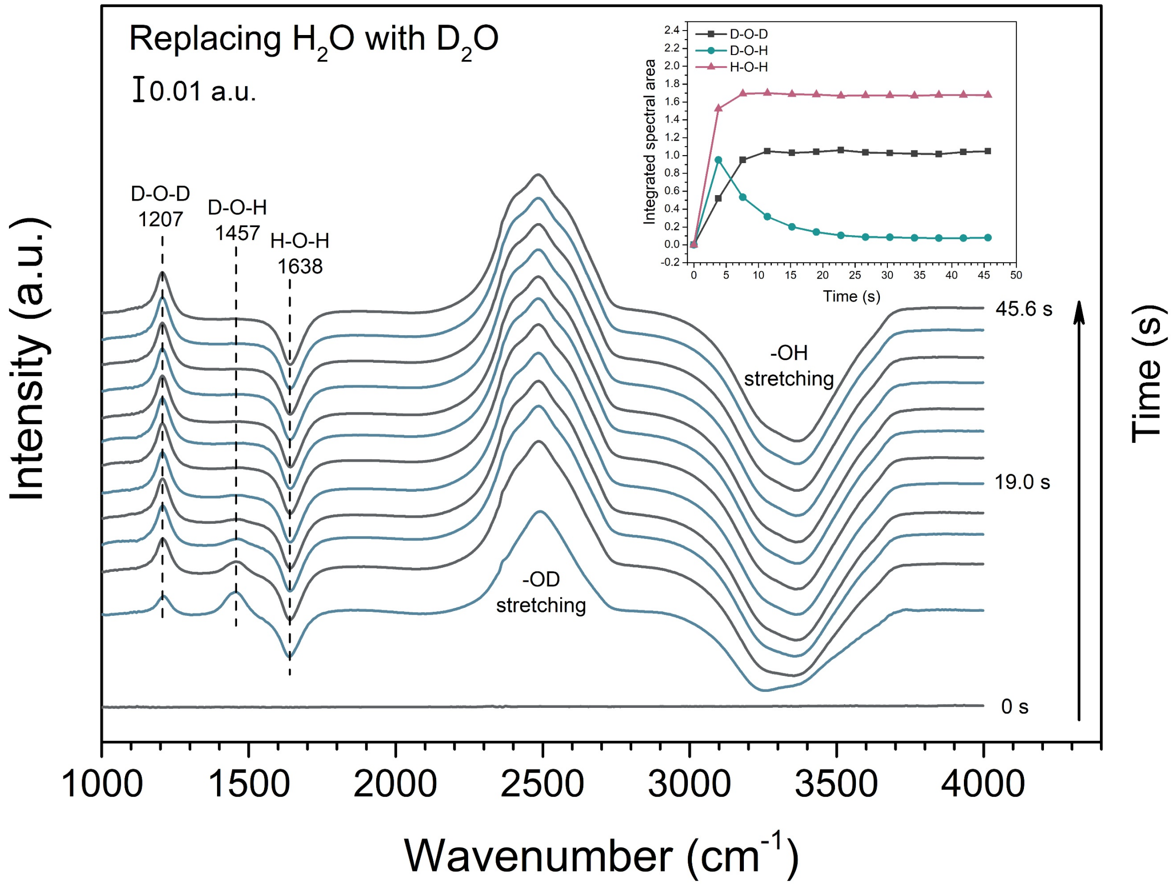

Supplementary Figure 8. Time evolution of the infrared bands that result from D-O-D, D-O-H, and H-O-H after replacing H_2O with D_2O. The background was taken in Ar-saturated H_2O before collecting the spectra. The inset shows the changes of the peak area of the D-O-D, D-O-H, and H-O-H band with time. Upon introducing the Ar-saturated D_2O, the signal of the D-O-D bending at 1207 cm^{-1} emerged and its intensity dramatically increased with time and plateaued in less than ten seconds (insert). We note that the increased intensity of H-O-H bending at 1638 cm^{-1} represents the desorption of adsorbed H_2O instead of adsorption due to its signal already been subtracted in background taking. Similar trend for the intensity change with time of OH and OD stretching in the region 2920 – 3740 cm^{-1} and 2120 – 2780 cm^{-1}, respectively, can also be observed. The decreased intensity with time of the band at 1457 cm^{-1} attributable to D-O-H bending originated from the reaction and equilibrium between H_2O and D_2O via transfer by self-dissociation and recombination to form HDO[1], represents the desorption of HDO which is slower than that of H_2O. These results indicate the H_2O can be thoroughly replaced with pulsed D_2O in a short period of time with less than ten seconds and thus enabled the reliable desorption rate measurement.

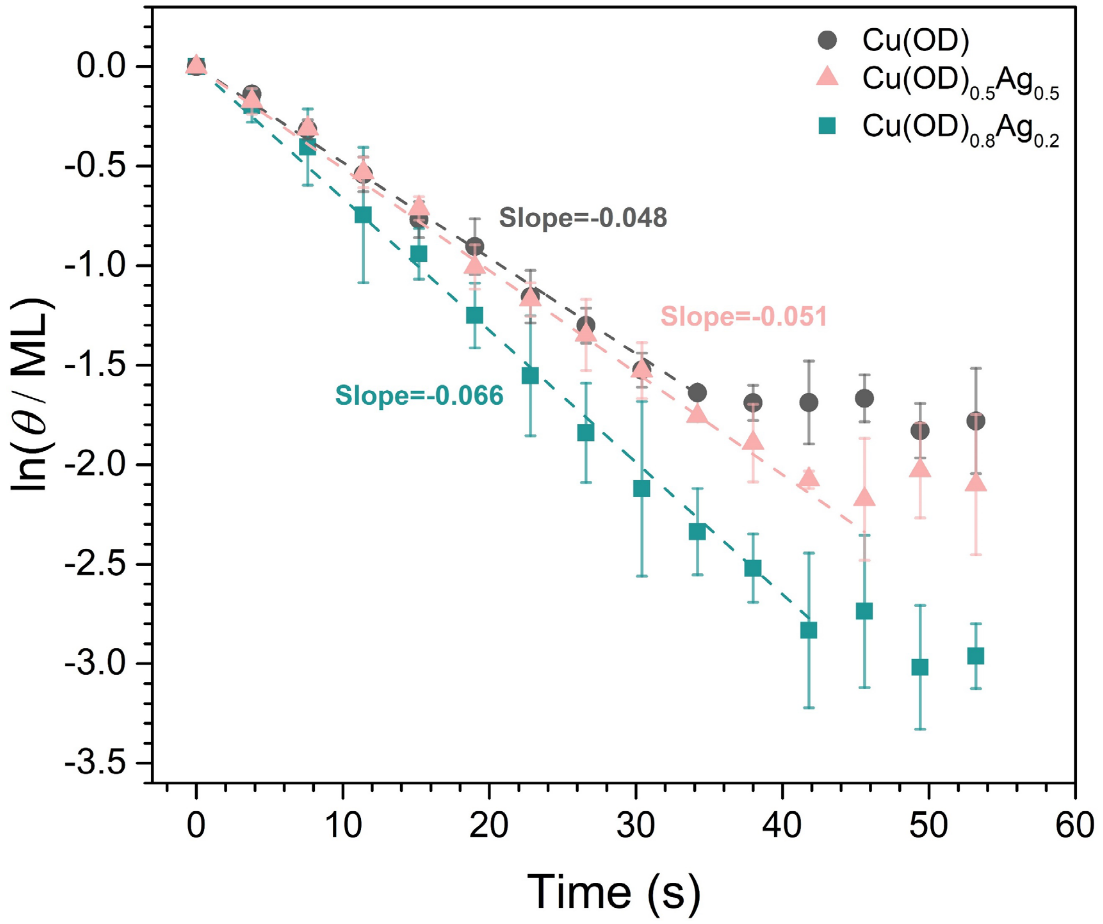

Supplementary Figure 9. Estimation of CO desorption rate constants on Cu(OD), Cu(OD)$_{0.5}$Ag$_{0.5}$, and Cu(OD)$_{0.8}$Ag$_{0.2}$ by fitting the first-order desorption rate expression. The error bars represent the standard deviation from at least three independent measurements.

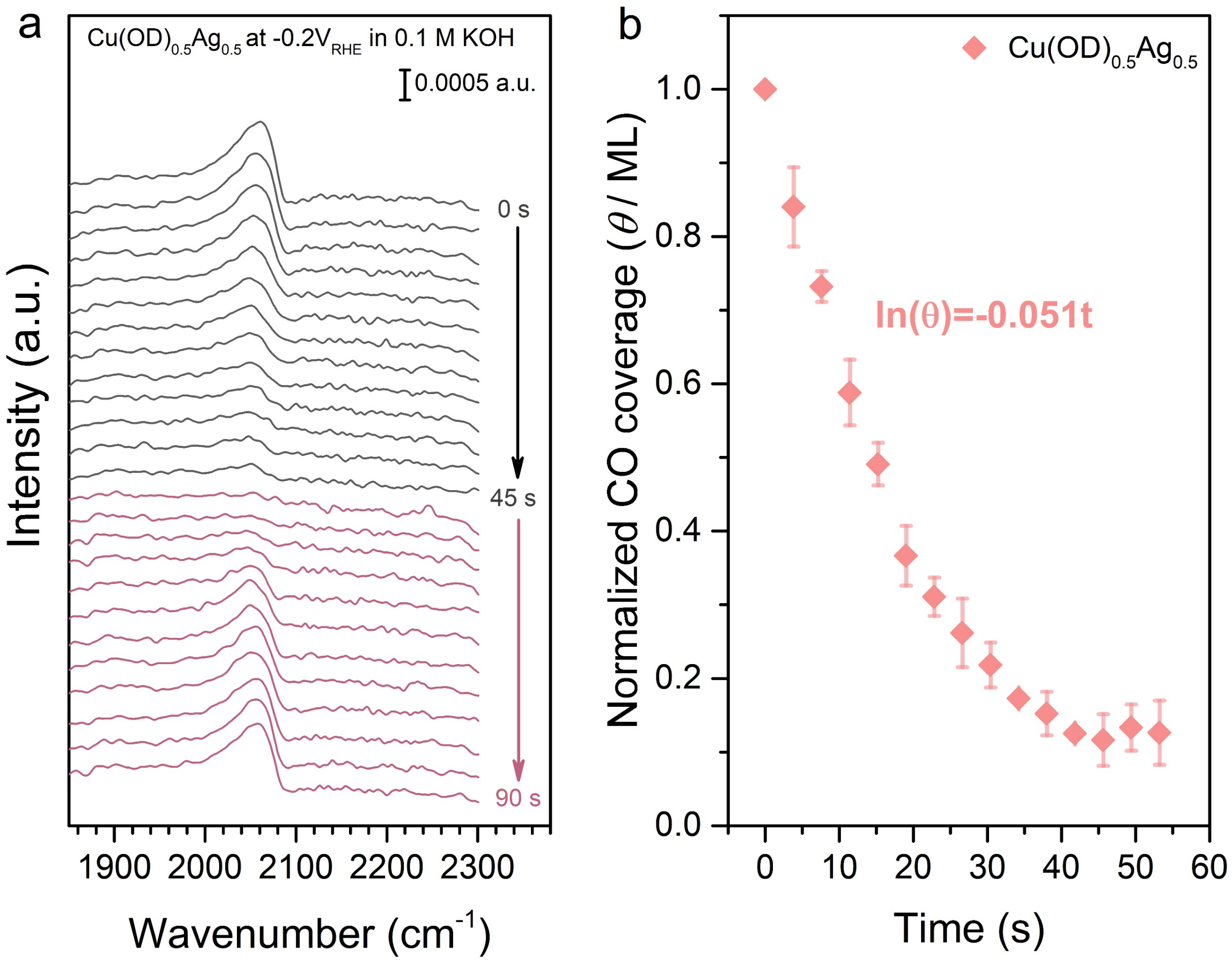

Supplementary Figure 10. Desorption rate of CO_{ad} measured by surface-enhanced infrared absorption spectroscopy on $Cu(OD)_{0.5}Ag_{0.5}$. (a) Representative time evolution of the infrared bands that result from CO bound to Cu surface recorded after removing CO in bulk solution by pulsing Ar saturated electrolyte and subsequently delivering CO saturated electrolyte at 45 s. (b) Normalized CO coverage obtained from the CO band in (a) as a function of time. The error bars represent standard deviation from at least three independent measurements.

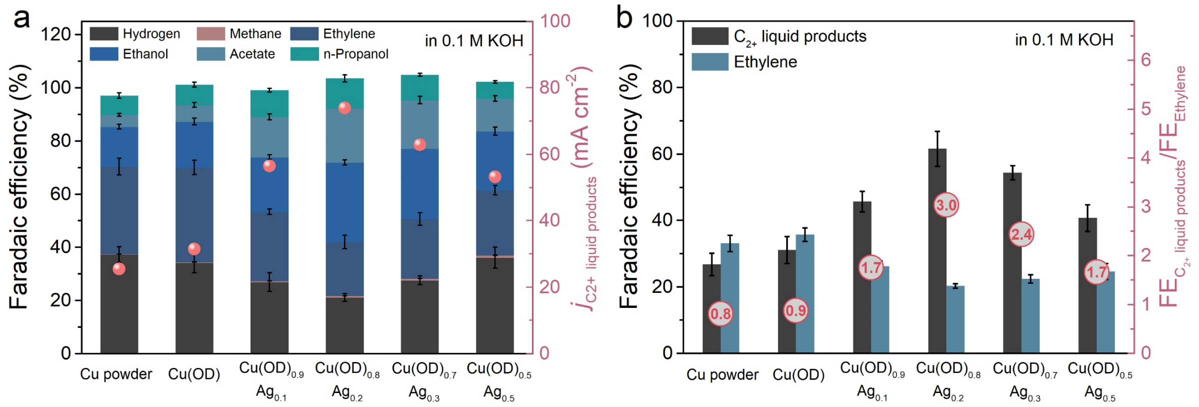

Supplementary Figure 11. CO electroreduction performance of different catalysts at -0.7 V in 0.1 M KOH. (a) Partial current density of C_{2+} liquid products and Faradaic efficiency of all CORR products. (b) FE_{C2+} liquid products versus $FE_{ethylene}$. Numbers in the red circles show the corresponding Faradaic efficiency ratio of C_{2+} liquid products to ethylene. The error bars represent the standard deviation from at least three independent measurements.

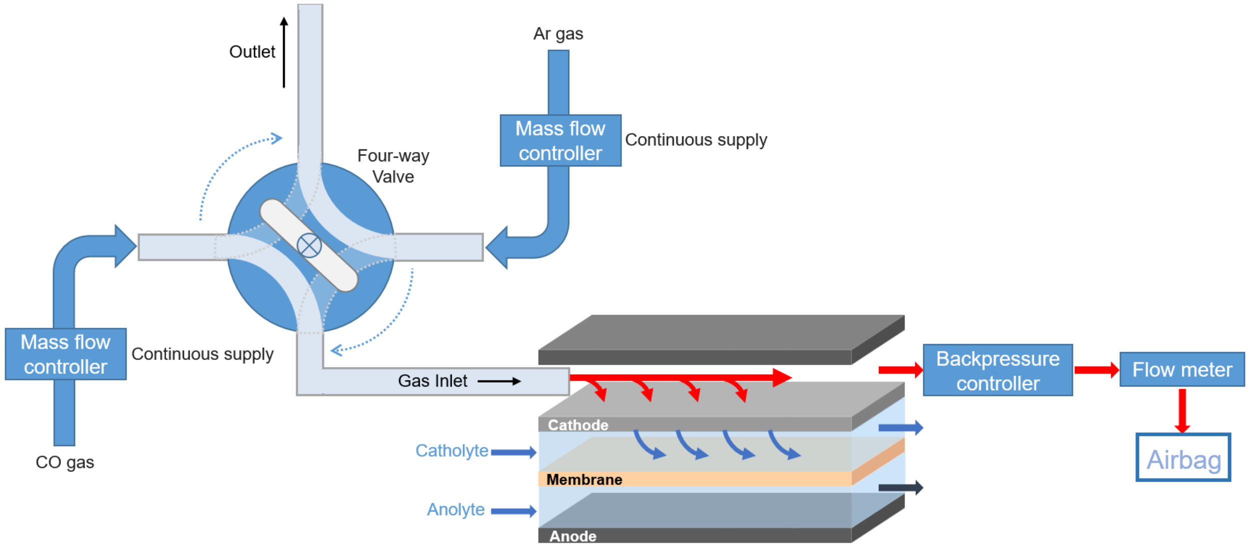

Supplementary Figure 12. A schematic of the gas switching experiments design. Two mass flow controllers were used to continuously flow CO and Ar gas at a steady flow rate into a four-way valve which delivers one gas feed to the flow cell and the other to the air. A backpressure controller and a flow meter were connected to the gas outlet of the flow cell to ensure the gas pressure and flow rate were not changed during gas switching.

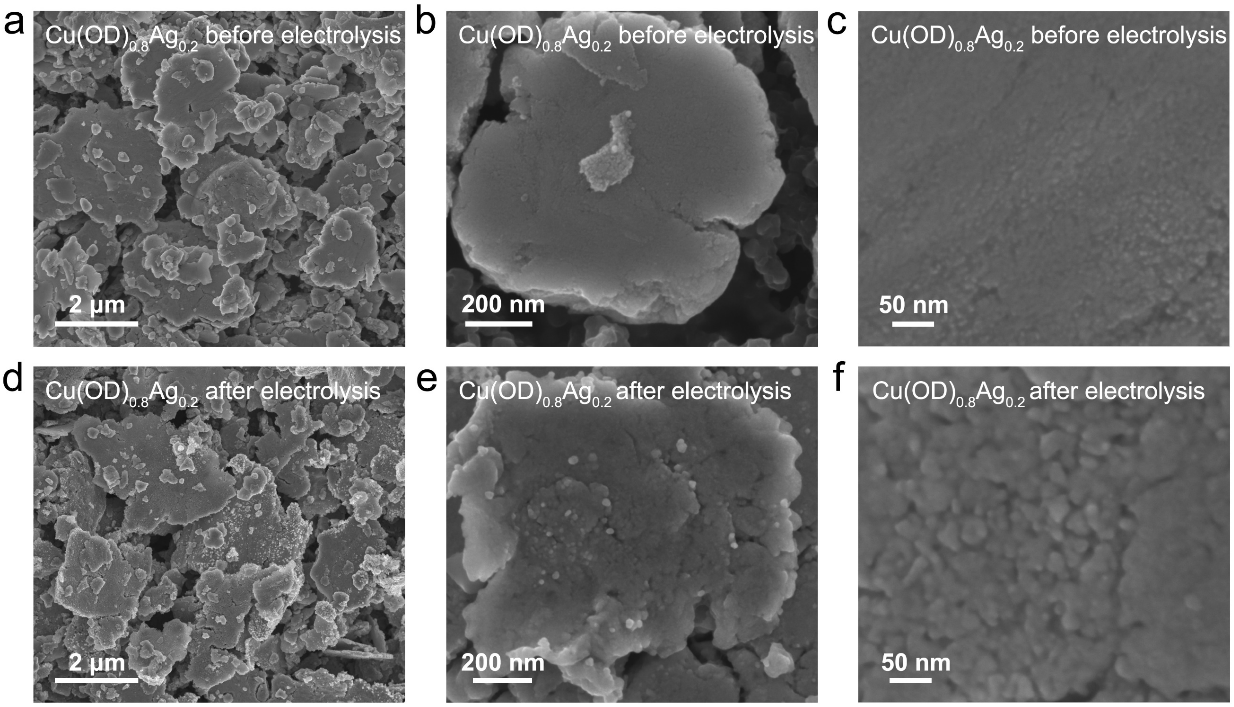

Supplementary Figure 13. Representative SEM images of $Cu(OD)_{0.8}Ag_{0.2}$ catalyst. SEM images of $Cu(OD)_{0.8}Ag_{0.2}$ catalyst (a-c) before and (d-f) after electrolysis, showing micro-plate morphology and no significant structural changes.

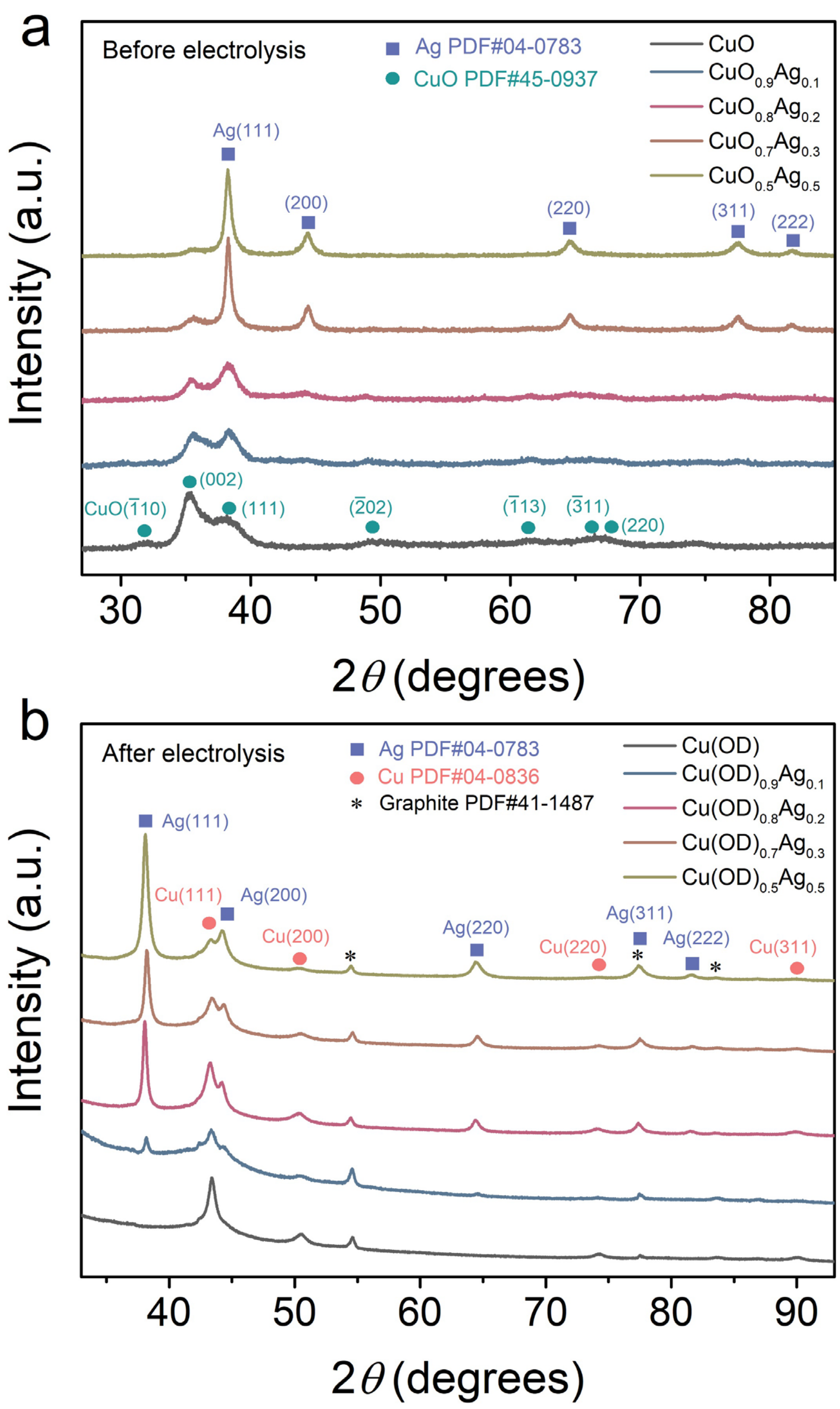

Supplementary Figure 14. Powder X-ray diffraction patterns of different catalysts. (a) XRD patterns of as-synthesized catalysts before electrolysis and (b) XRD patterns of the corresponding electrodes after electrolysis.

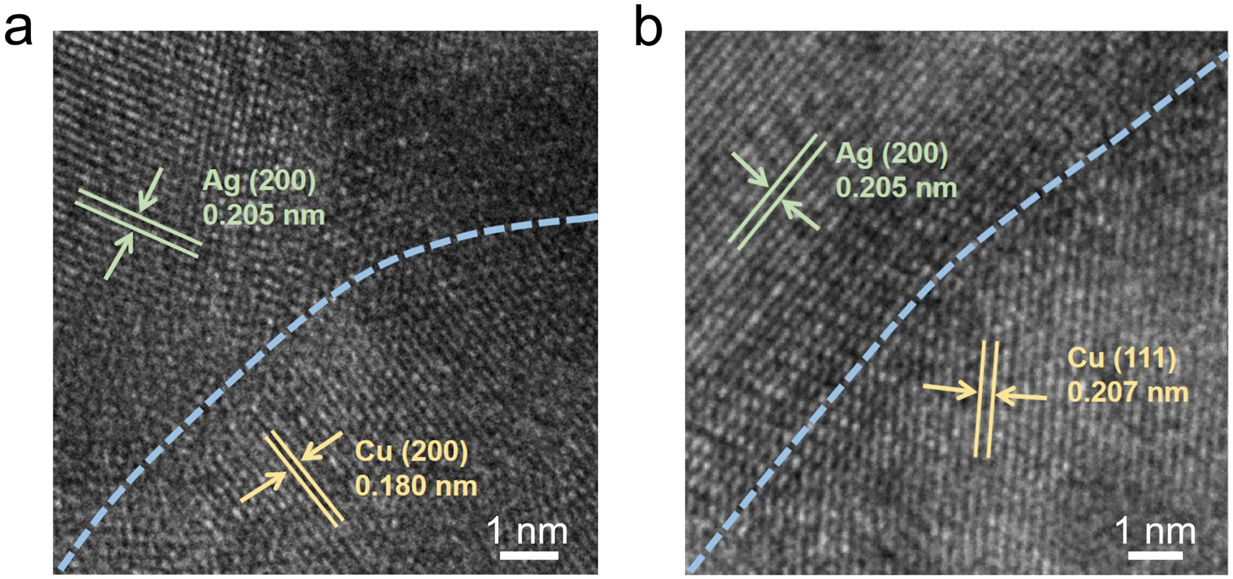

Supplementary Figure 15. Electron diffraction pattern and HRTEM image of $Cu(OD)_{0.8}Ag_{0.2}$. HRTEM image of Cu-Ag phase boundaries with different orientations including (a) fcc Ag (200) planes and fcc Cu (200) planes and (b) fcc Ag (200) planes and fcc Cu (111) planes.

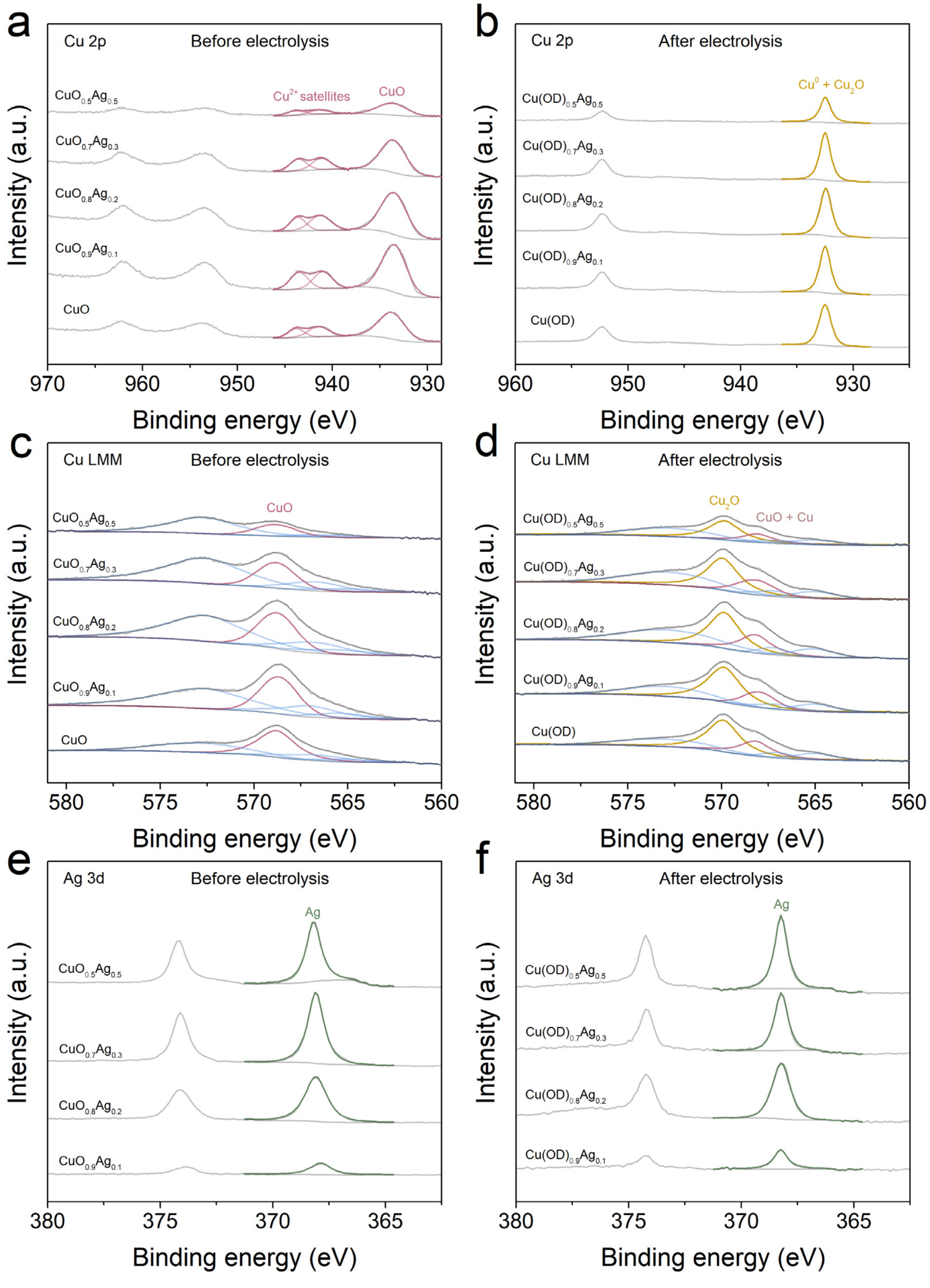

Supplementary Figure 16. X-ray photoelectron spectroscopy characterizations for different catalysts. X-ray photoelectron spectra of the (a, b) Cu 2p peaks, (c, d) the Cu LMM region and (e, f) Ag 3d peaks before and after electrolysis. The three additional peaks (bule) in the Cu LMM spectra located at approximately 572.8, 567.0 and 565.0 eV are due to different transition states[2,3].

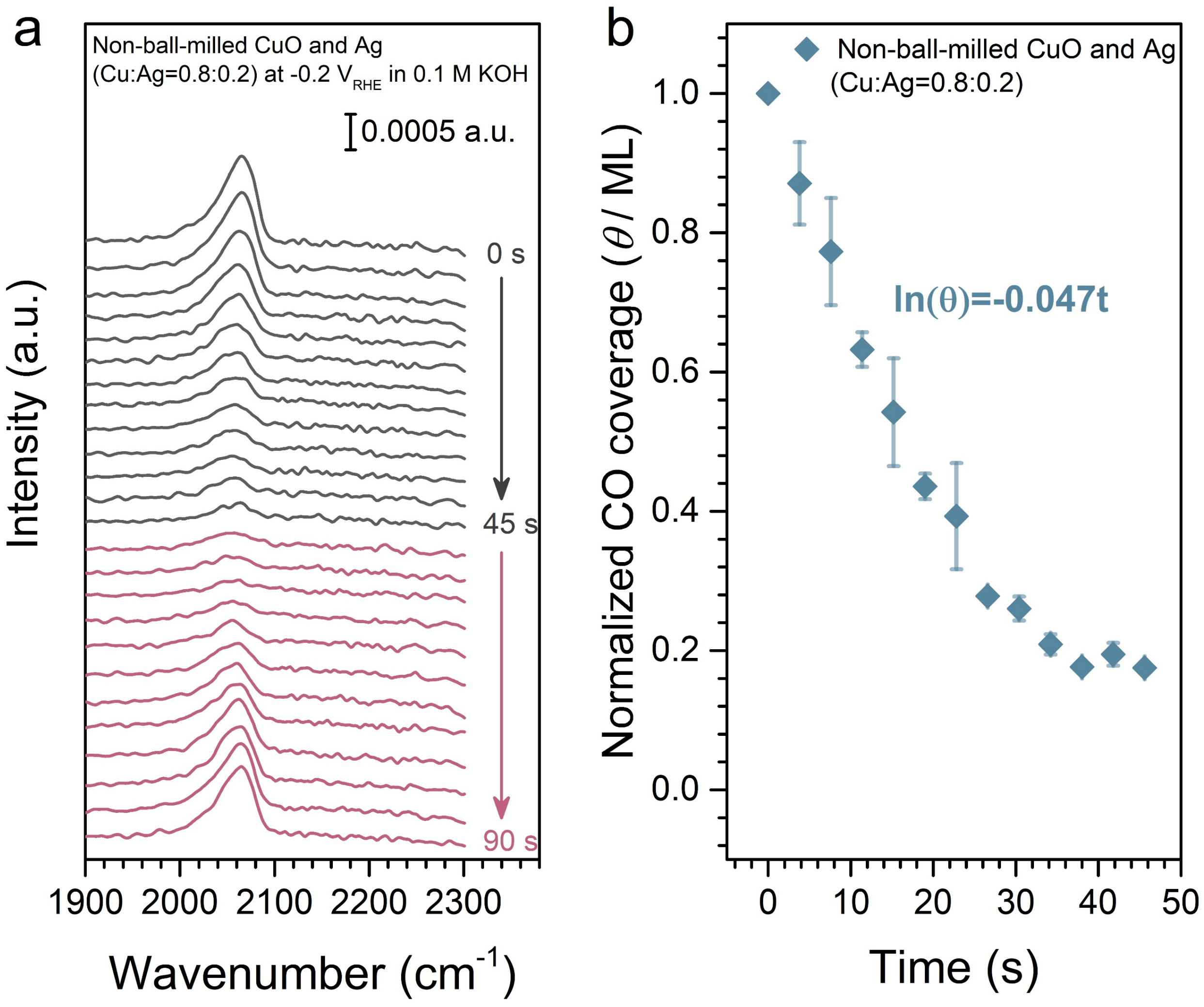

Supplementary Figure 17. Desorption rate of CO_{ad} measured by surface-enhanced infrared absorption spectroscopy on the non-ball-milled CuO and Ag powder mixture (Cu:Ag=0.8:0.2). (a) Representative time evolution of the infrared bands that result from CO bound to Cu surface recorded after removing CO in bulk solution by pulsing Ar saturated electrolyte and subsequently delivering CO saturated electrolyte at 45s. (b) Normalized CO coverage obtained from the CO band in (a) as a function of time. The error bars represent standard deviation from at least three independent measurements.

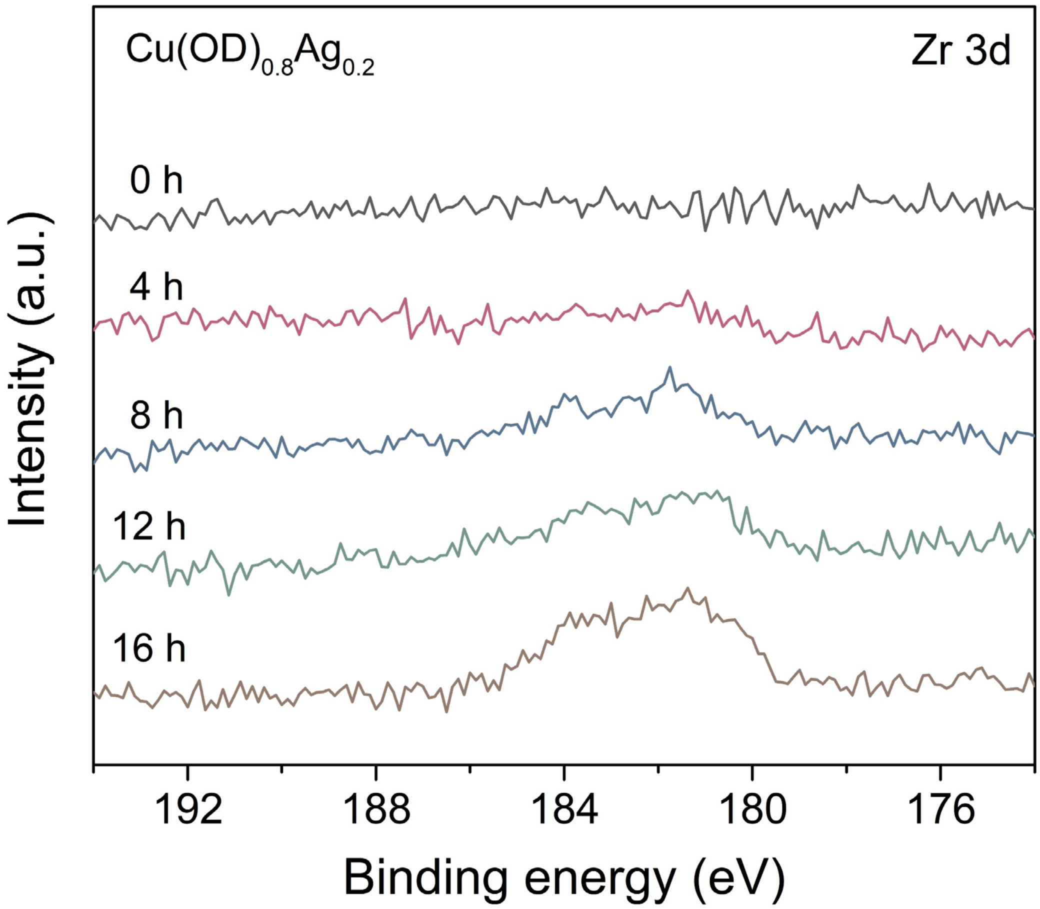

Supplementary Figure 18. X-ray photoelectron spectra of Zr 3d for $Cu(OD)_{0.8}Ag_{0.2}$ catalysts with different ball milling time.

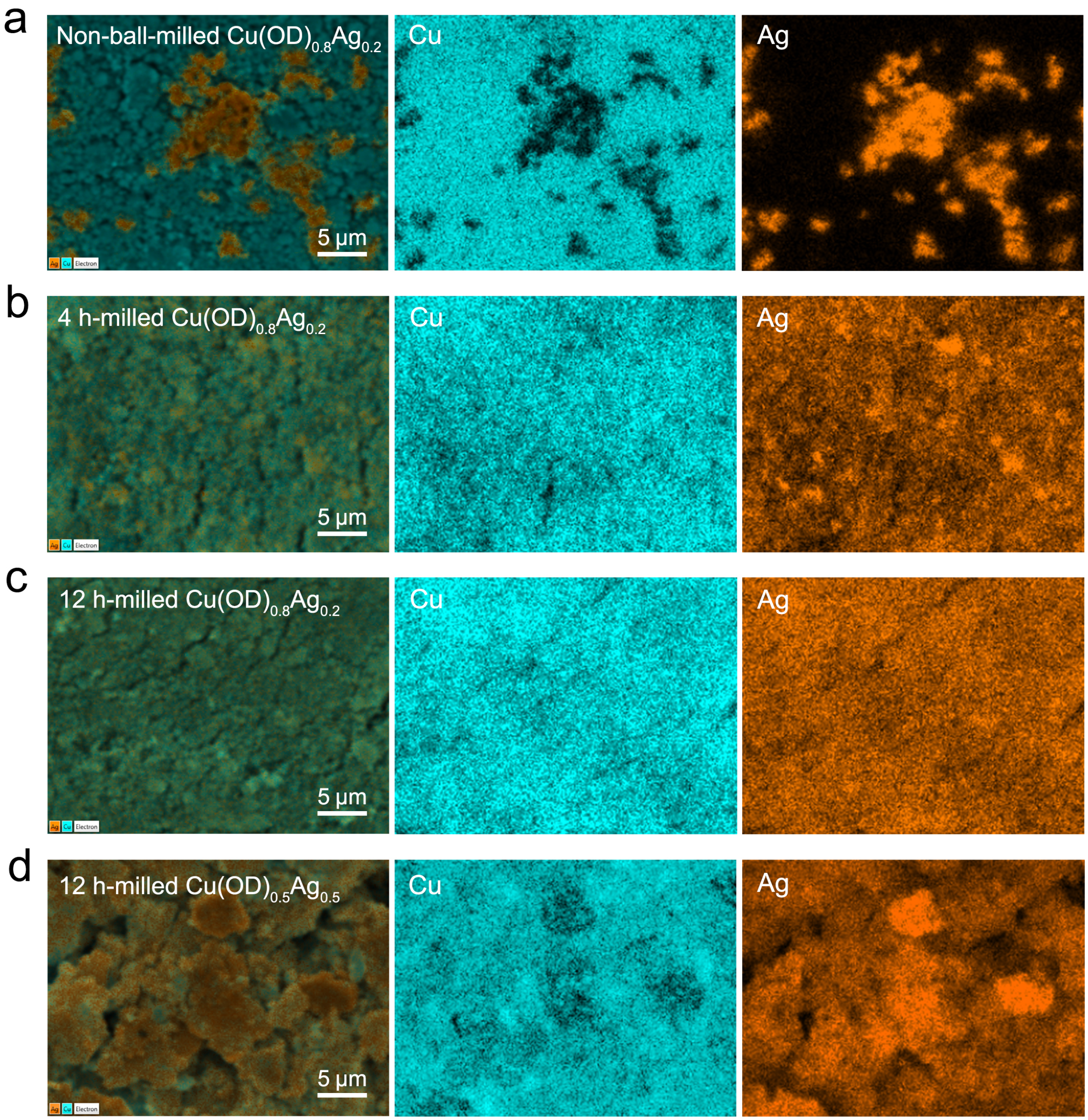

Supplementary Figure 19. SEM-EDS elemental mapping of $Cu(OD)_{0.8}Ag_{0.2}$ and $Cu(OD)_{0.5}Ag_{0.5}$ catalysts. EDS elemental mappings of (a) non-ball-milled, (b) 4 h-milled and (c) 12 h-milled CuO and Ag powder (Cu:Ag=0.8:0.2), which indicates the homogeneity of CuO and Ag mixture improves with extended ball milling time. (d) EDS elemental mappings of 12 h-milled CuO and Ag powder (Cu:Ag=0.5:0.5), which shows that further increasing Ag content decreases the homogeneity of CuO and Ag mixture.

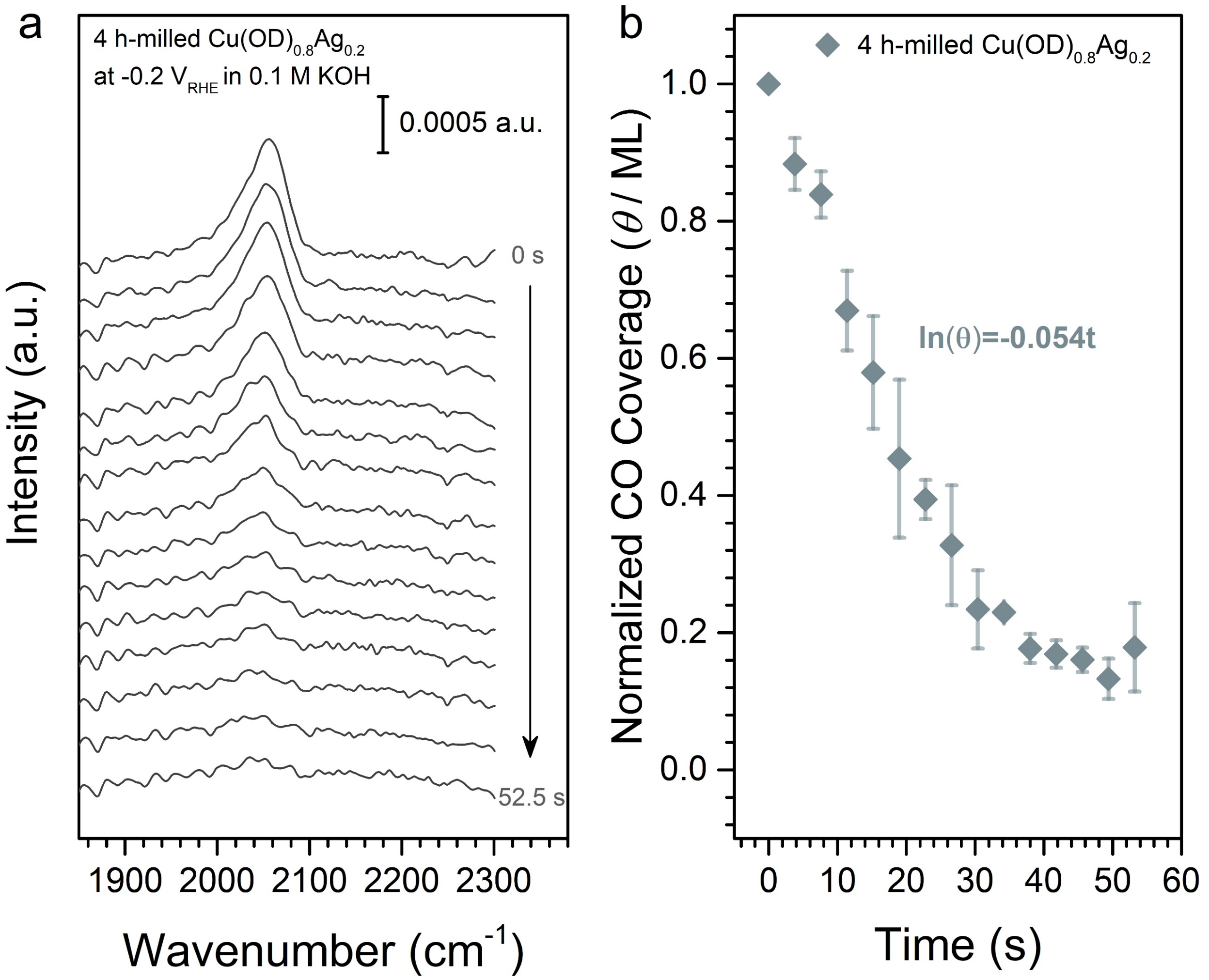

Supplementary Figure 20. Desorption rate of CO_{ad} measured by surface-enhanced infrared absorption spectroscopy on 4 h-milled $Cu(OD)_{0.8}Ag_{0.2}$. (a) Representative time evolution of the infrared bands that result from CO bound to Cu surface recorded after removing CO in bulk solution by pulsing Ar saturated electrolyte. (b) Normalized CO coverage obtained from the CO band in (a) as a function of time. The error bars represent standard deviation from at least three independent measurements.

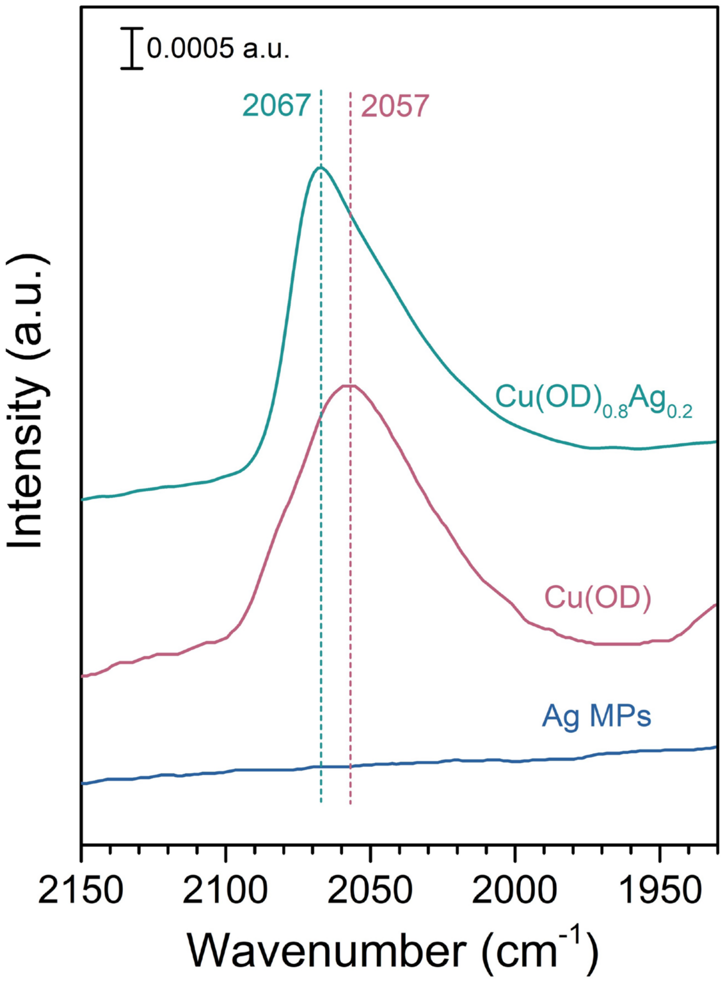

Supplementary Figure 21. Linearly adsorbed CO band on $Cu(OD)_{0.8}Ag_{0.2}$, Cu(OD), and pure Ag micro-sized particles (Ag MPs).

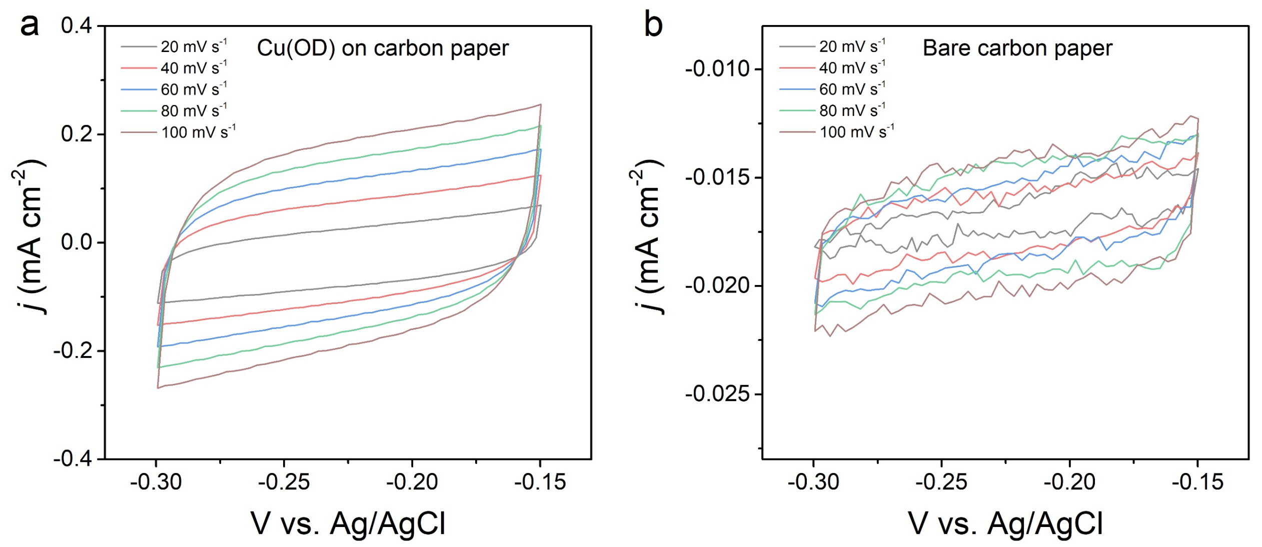

Supplementary Figure 22. ECSA measurements for (a) the carbon paper supported Cu(OD) electrode and (b) the bare carbon paper electrode.

Supplementary Table 1. Comparison of C_{2+} liquid products Faradaic efficiencies (FE) and $FE_{C2+\ liquid\ products}/FE_{ethylene}$ of different catalysts for CO and CO_2 electroreduction operated at commercially relevant current densities (> 100 mA cm^{-2}).

Catalyst	Feed-stock	Electrolyte	Potential (V_{RHE})	$FE_{C2+\ liquids}$	$FE_{Ethylene}$	$FE_{C2+\ liquid}/FE_{ethylene}$ ratio	Ref.
**$Cu(OD)_{0.8}Ag_{0.2}$**	**CO**	**1 M KOH**	**-0.56**	**76.9**	**13.3**	**5.8**	**This work**
OD-Cu	CO	1 M KOH	-0.60	42.6	37.5	1.1	4
Micron-sized Cu	CO	1 M KOH	-0.65	46.1	32.7	1.4	
$Cu_{0.9}Ag_{0.1}$	CO	1 M KOH	-0.77	48.7	12.7	3.8	5
$Cu_{0.9}Ni_{0.1}$	CO	1 M KOH	-0.76	54.6	10.4	5.2	
Cu nanosheets	CO	1 M KOH	-0.76	41.8	19.1	2.2	6
Cu-HDD	CO	1 M KOH	-0.76	57[a]	34[a]	1.7	7
Cu nanocubes	CO	1 M KOH	$-2.24_{IR\ free}$	45[a]	18[a]	2.5	8
Cu cavity I	CO	1 M KOH	-0.56	37.6	27.5	1.4	9
Cu cavity II	CO	1 M KOH	-0.56	41.3	21	2.0	
Ag-doped Cu	CO	1 M KOH	-0.46	43.9	29.7	1.5	10
$CuPd_{0.007}$	CO	1 M KOH	-0.62	54.4	37	1.5	11
Cu nanoparticles	CO	1 M KOH	-0.69	36.4	55.5	0.6	
CuPd	CO	1 M KOH	-0.79	66	21	3.1	12
NGQ/Cu-nr	CO	1 M KOH	-0.70	88[a]	9[a]	9.8	13
Cu-wire	CO_2	1 M KOH	-0.60	32	38.2	0.8	14
CuAg-wire	CO_2	1 M KOH	-0.68	55.2	29.9	1.8	
CuPb-0.7/C	CO_2	1 M KOH	N.A.	49.5	24.5	2.0	15
$Ag_{0.14}Cu_{0.86}$	CO_2	1 M KOH	-0.67	48[a]	35[a]	1.4	16
Cu/FeTPP[Cl]	CO_2	1 M $KHCO_3$	-0.82	47[a]	38[a]	1.2	17
34% N-C/Cu	CO_2	1 M KOH	-0.68	56.0	37.5	1.5	18
$Ce(OH)_x/Cu/PTFE$	CO_2	1 M KOH	-0.70	46.5	33.8	1.4	19

[a]This value is digitized from the graphical results in the reference.

Supplementary Table 2. Calculated double layer capacitance of each catalyst and corresponding roughness factor.

Electrode	C_{DL} (mF cm^{-2})	Roughness Factor for Both Cu and Ag	Roughness Factor for Cu
Cu(OD)	1.51	52.1	52.1
$Cu(OD)_{0.9}Ag_{0.1}$	1.64	55.2	54.7
$Cu(OD)_{0.8}Ag_{0.2}$	1.68	56.1	55.5
$Cu(OD)_{0.7}Ag_{0.3}$	1.71	55.7	54.5
$Cu(OD)_{0.5}Ag_{0.5}$	2.05	62.0	59.0
Ag Foil	0.11	1.0	
Cu Foil[20]	0.029	1.0	1.0

Supplementary Table 3. Atomic percentage of Cu and Ag for each catalyst obtained from XPS data.

Electrode	Before electrolysis		After electrolysis	
	Ag%	Cu%	Ag%	Cu%
Cu(OD)	0	100	0	100
$Cu(OD)_{0.9}Ag_{0.1}$	4.4%	95.6%	3.2%	96.8%
$Cu(OD)_{0.8}Ag_{0.2}$	22.4%	77.6%	4.2%	95.8%
$Cu(OD)_{0.7}Ag_{0.3}$	27.5%	72.5%	7.6%	92.4%
$Cu(OD)_{0.5}Ag_{0.5}$	47.0%	53.0%	16.6%	83.4%

References

(1) Belhadj, H.; Hakki, A.; Robertson, P. K. & Bahnemann, D. W. In situ ATR-FTIR study of H_2O and D_2O adsorption on TiO_2 under UV irradiation. *Phys. Chem. Chem. Phys.* **17**, 22940-6 (2015).
(2) Platzman, I.; Brener, R.; Haick, H. & Tannenbaum, R. Oxidation of polycrystalline copper thin films at ambient conditions. *J. Phys. Chem. C* **112**, 1101-1108 (2008).
(3) Dubot, P.; Jousset, D.; Pinet, V.; Pellerin, F. & Langeron, J. P. Simulation of the Lmm Auger-Spectra of Copper. *Surf. Interface Anal.* **12**, 99-104 (1988).
(4) Jouny, M.; Luc, W. & Jiao, F. High-rate electroreduction of carbon monoxide to multi-carbon products. *Nat. Catal.* **1**, 748-755 (2018).
(5) Yang, C.; Ko, B. H.; Hwang, S.; Liu, Z.; Yao, Y.; Luc, W., et al. Overcoming immiscibility toward bimetallic catalyst library. *Sci. Adv.* **6**, eaaz6844 (2020).
(6) Luc, W.; Fu, X. B.; Shi, J. J.; Lv, J. J.; Jouny, M.; Ko, B. H., et al. Two-dimensional copper nanosheets for electrochemical reduction of carbon monoxide to acetate. *Nat. Catal.* **2**, 423-430 (2019).
(7) Zhao, C.; Luo, G.; Liu, X.; Zhang, W.; Li, Z.; Xu, Q., et al. In Situ Topotactic Transformation of an Interstitial Alloy for CO Electroreduction. *Adv. Mater.* **32**, e2002382 (2020).
(8) Zhu, P.; Xia, C.; Liu, C. Y.; Jiang, K.; Gao, G.; Zhang, X., et al. Direct and continuous generation of pure acetic acid solutions via electrocatalytic carbon monoxide reduction. *Proc. Natl. Acad. Sci. U. S. A.* **118**, e2010868118 (2021).
(9) Zhuang, T.-T.; Pang, Y.; Liang, Z.-Q.; Wang, Z.; Li, Y.; Tan, C.-S., et al. Copper nanocavities confine intermediates for efficient electrosynthesis of C3 alcohol fuels from carbon monoxide. *Nat. Catal.* **1**, 946-951 (2018).
(10) Wang, X.; Wang, Z.; Zhuang, T. T.; Dinh, C. T.; Li, J.; Nam, D. H., et al. Efficient upgrading of CO to C_3 fuel using asymmetric C-C coupling active sites. *Nat. Commun.* **10**, 5186 (2019).
(11) Li, J.; Xu, A.; Li, F.; Wang, Z.; Zou, C.; Gabardo, C. M., et al. Enhanced multi-carbon alcohol electroproduction from CO via modulated hydrogen adsorption. *Nat. Commun.* **11**, 3685 (2020).
(12) Ji, Y. L.; Chen, Z.; Wei, R. L.; Yang, C.; Wang, Y. H.; Xu, J., et al. Selective CO-to-acetate electroreduction via intermediate adsorption tuning on ordered Cu-Pd sites. *Nat. Catal.* **5**, 251-258 (2022).
(13) Chen, C.; Yan, X.; Liu, S.; Wu, Y.; Wan, Q.; Sun, X., et al. Highly Efficient Electroreduction of CO_2 to C_{2+} Alcohols on Heterogeneous Dual Active Sites. *Angew. Chem. Int. Ed. Engl.* **59**, 16459-16464 (2020).
(14) Hoang, T. T. H.; Verma, S.; Ma, S.; Fister, T. T.; Timoshenko, J.; Frenkel, A. I., et al. Nanoporous Copper-Silver Alloys by Additive-Controlled Electrodeposition for the Selective Electroreduction of CO_2 to Ethylene and Ethanol. *J. Am. Chem. Soc.* **140**, 5791-5797 (2018).
(15) Wang, P.; Yang, H.; Xu, Y.; Huang, X.; Wang, J.; Zhong, M., et al. Synergized Cu/Pb Core/Shell Electrocatalyst for High-Efficiency CO_2 Reduction to C_{2+} Liquids. *ACS Nano* **15**, 1039-1047 (2021).
(16) Li, Y. C.; Wang, Z.; Yuan, T.; Nam, D. H.; Luo, M.; Wicks, J., et al. Binding Site Diversity Promotes CO_2 Electroreduction to Ethanol. *J. Am. Chem. Soc.* **141**, 8584-8591 (2019).
(17) Li, F.; Li, Y. C.; Wang, Z.; Li, J.; Nam, D.-H.; Lum, Y., et al. Cooperative CO_2-to-ethanol conversion via enriched intermediates at molecule–metal catalyst interfaces. *Nat. Catal.* **3**, 75-82 (2019).
(18) Wang, X.; Wang, Z.; García de Arquer, F. P.; Dinh, C.-T.; Ozden, A.; Li, Y. C., et al. Efficient electrically powered CO_2-to-ethanol via suppression of deoxygenation. *Nat. Energy* **5**, 478-486 (2020).
(19) Luo, M.; Wang, Z.; Li, Y. C.; Li, J.; Li, F.; Lum, Y., et al. Hydroxide promotes carbon dioxide electroreduction to ethanol on copper via tuning of adsorbed hydrogen. *Nat. Commun.* **10**, 5814 (2019).
(20) Li, C. W.; Ciston, J. & Kanan, M. W. Electroreduction of carbon monoxide to liquid fuel on oxide-derived nanocrystalline copper. *Nature* **508**, 504-507 (2014).
